# Reduced RBPMS Levels Promote Cell Proliferation and Decrease Cisplatin Sensitivity in Ovarian Cancer Cells

**DOI:** 10.3390/ijms23010535

**Published:** 2022-01-04

**Authors:** Robert J. Rabelo-Fernández, Ginette S. Santiago-Sánchez, Rohit K. Sharma, Abiel Roche-Lima, Kelvin Carrasquillo Carrion, Ricardo A. Noriega Rivera, Blanca I. Quiñones-Díaz, Swetha Rajasekaran, Jalal Siddiqui, Wayne Miles, Yasmarie Santana Rivera, Fatima Valiyeva, Pablo E. Vivas-Mejia

**Affiliations:** 1 University of Puerto Rico Comprehensive Cancer Center, San Juan, PR 00935, USA; robertrabelo1@gmail.com (R.J.R.-F.); ginette.santiago@upr.edu (G.S.S.-S.); rohit.sharma@berkeley.edu (R.K.S.); ricardo.noriega1@upr.edu (R.A.N.R.); blanca.quinones@upr.edu (B.I.Q.-D.); fvaliyeva@cccupr.org (F.V.); 2 Department of Biology, University of Puerto Rico, Rio Piedras Campus, San Juan, PR 00925, USA; 3 Department of Biochemistry, University of Puerto Rico, Medical Sciences Campus, San Juan, PR 00935, USA; 4 Deanship of Research, University of Puerto Rico, Medical Sciences Campus, San Juan, PR 00935, USA; abiel.roche@upr.edu (A.R.-L.); kelvin.carrasquilllo@upr.edu (K.C.C.); 5 Department of Cancer Biology and Genetics, Ohio State University Comprehensive Cancer Center, Columbus, OH 43210, USA; rajasekaran.11@osu.edu (S.R.); Jalal@osumc.edu (J.S.); Wayne.Miles@osumc.edu (W.M.); 6 School of Dentistry, University of Puerto Rico, Medical Sciences Campus, San Juan, PR 00935, USA; yasmarie.santana@upr.edu

**Keywords:** RNA binding protein with multiple splicing, ovarian cancer, CRISPR, cisplatin resistance, tumor suppressor gene

## Abstract

Worldwide, the number of cancer-related deaths continues to increase due to the ability of cancer cells to become chemotherapy-resistant and metastasize. For women with ovarian cancer, a staggering 70% will become resistant to the front-line therapy, cisplatin. Although many mechanisms of cisplatin resistance have been proposed, the key mechanisms of such resistance remain elusive. The RNA binding protein with multiple splicing (RBPMS) binds to nascent RNA transcripts and regulates splicing, transport, localization, and stability. Evidence indicates that RBPMS also binds to protein members of the AP-1 transcription factor complex repressing its activity. Until now, little has been known about the biological function of RBPMS in ovarian cancer. Accordingly, we interrogated available Internet databases and found that ovarian cancer patients with high RBPMS levels live longer compared to patients with low RBPMS levels. Similarly, immunohistochemical (IHC) analysis in a tissue array of ovarian cancer patient samples showed that serous ovarian cancer tissues showed weaker RBPMS staining when compared with normal ovarian tissues. We generated clustered regularly interspaced short palindromic repeats (CRISPR)-mediated RBPMS knockout vectors that were stably transfected in the high-grade serous ovarian cancer cell line, OVCAR3. The knockout of RBPMS in these cells was confirmed via bioinformatics analysis, real-time PCR, and Western blot analysis. We found that the RBPMS knockout clones grew faster and had increased invasiveness than the control CRISPR clones. RBPMS knockout also reduced the sensitivity of the OVCAR3 cells to cisplatin treatment. Moreover, β-galactosidase (β-Gal) measurements showed that RBPMS knockdown induced senescence in ovarian cancer cells. We performed RNAseq in the RBPMS knockout clones and identified several downstream-RBPMS transcripts, including non-coding RNAs (ncRNAs) and protein-coding genes associated with alteration of the tumor microenvironment as well as those with oncogenic or tumor suppressor capabilities. Moreover, proteomic studies confirmed that RBPMS regulates the expression of proteins involved in cell detoxification, RNA processing, and cytoskeleton network and cell integrity. Interrogation of the Kaplan–Meier (KM) plotter database identified multiple downstream-RBPMS effectors that could be used as prognostic and response-to-therapy biomarkers in ovarian cancer. These studies suggest that RBPMS acts as a tumor suppressor gene and that lower levels of RBPMS promote the cisplatin resistance of ovarian cancer cells.

## 1. Introduction

Ovarian cancer remains the most lethal gynecological malignancy in the United States [1,2]. The American Cancer Society estimates about 21,410 new cases of ovarian cancer will be diagnosed in the United State in 2021, of which 13,770 (>60%) patients will die of the disease [2]. This high death rate stems from most ovarian cancer patients not being diagnosed until an advanced stage. Approximately 90% of ovarian cancers are malignant epithelial ovarian cancers (EOCs) [3]. EOC is a heterogeneous disease comprised of five histological subtypes: high-grade serous, low-grade serous, mucinous, endometrioid, and clear cell tumors [4]. High-grade serous carcinomas (HGSCs) account for 70% of tumor types [5,6]. Although most ovarian cancer patients respond to standard treatment, which is based on a combination of cytoreductive surgery and platinum/taxane chemotherapy, relapse occurs in over 60% of treated patients, resulting in chemoresistant fatal disease [7].

Postulated mechanisms of cisplatin resistance include decreased levels of the receptors/channels that reduce the influx of cisplatin inside the cells, increased levels of proteins/channels that promote cisplatin efflux, increased intracellular levels of specific sulfur-containing macromolecules that reduce the nuclear net cisplatin concentration, the deregulation of DNA repair mechanisms, and metabolic rewiring that confers growth advantages to particular cell populations [8]. Additionally, studies have indicated that the inactivation of intrinsic cell death pathways [9,10]; the activation of cell survival pathways [11]; and the dysregulation of oncogenes [12], tumor suppressor genes [13], and non-coding RNAs [14] also play a central role in the cisplatin resistance of cancer cells.

Previously, we reported that the RNA binding protein with multiple splicing (RBPMS) is a miR-21-3p target gene in cisplatin-resistant ovarian cancer cells [15]. RBPMS—also known as HERMES [16,17]—is a gene located on chromosome 8 position p12 spanning over 230 kb (30,241,924 to 30,430,508 bp) in the human genome [18]. RBPMS is characterized by its possession of a single RNA recognition motif (RRM), which corresponds to a protein domain of ~80 amino acids (AA) flanked by 23 AA N-terminal and 95 AA C-terminal regions. Reports have indicated that RBPMS is expressed at high levels in the heart, breasts, lungs, kidneys, stomach, muscles, liver, eyes, adipose tissue, and ovaries [19,20,21,22,23]. Fu and colleagues reported that the reduced expression of RBPMS promotes the proliferation and migration of breast cancer cells [21]. However, the biological role of RBPMS in ovarian cancer and/or the cisplatin resistance of ovarian cancer cells is unknown. Using Internet search tools and an ovarian cancer tissue array, we studied correlations between RBPMS expression levels and ovarian cancer patient outcomes. Then, we investigated the biological effects of CRISPR/Cas9-mediated RBPMS knockout in OVCAR on cell viability, proliferation, and invasion capacity. In addition, we performed RNAseq and proteomics experiments in RBPMS knockout clones, finding that RBPMS regulates many non-coding RNAs (ncRNAs) and protein-coding genes associated with alteration of the tumor microenvironment, cell detoxification, RNA processing, cytoskeleton and cell integrity. Interrogation of the KM plotter database (https://kmplot.com, accessed on 12 October 2021) revealed that multiple downstream-RBPMS effectors correlate well with the overall survival (OS) and progression-free survival (PFS) of the disease. Overall, our findings provide new evidence indicating that reduced levels of RBPMS contribute to the cell growth and invasion as well as drug resistance of ovarian cancer cells as well as that RBPMS could act as a tumor suppressor gene in these cells.

## 2. Results

### 2.1. RBPMS Protein Levels Are Reduced in Ovarian Cancer Tumor Samples and Correlate with Poor Prognosis

**Figure 1:**
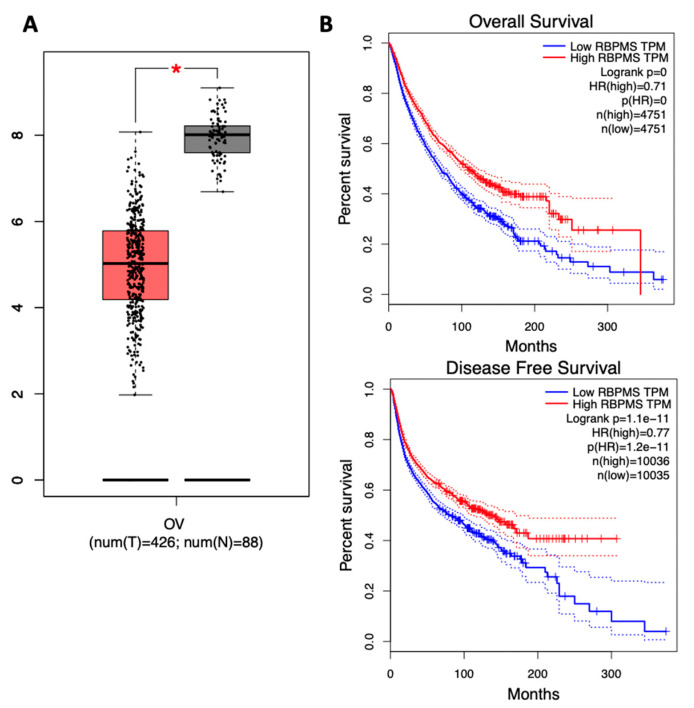

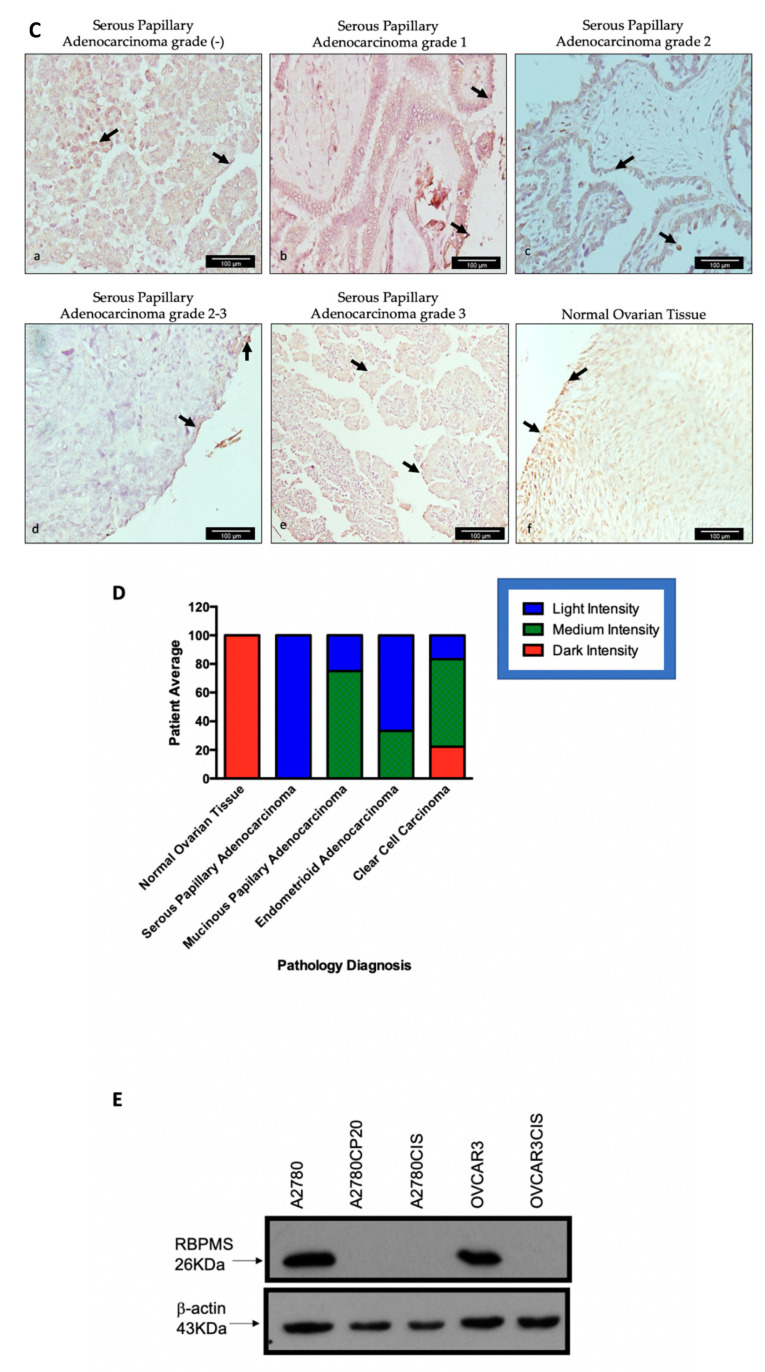
**RBPMS protein levels are reduced in ovarian cancer tumor samples and correlate with poor prognosis:** (**A**) Relative expression levels of RBPMS in ovarian cancer tumor tissues and normal ovarian tissues were analyzed using the GEPIA bioinformatic tool. * *p* < 0.05. The red box represents the cancer tissue samples, while the black box represents the normal tissue samples. (**B**) Survival plots of the ovarian cancer patients were generated using the Kaplan–Meier (KM) plotter. Overall survival (OS) and disease-free survival (DFS) of patients with ovarian cancer stratified by expression levels of RBPMS are shown based on RNA-Seq data (graphs generated automatically using GEPIA). (**C**) Representative images of IHC analysis of the tissue array. (**a**) Non-assigned grade (-) serous papillary adenocarcinoma. (**b**) grade 1 serous papillary adenocarcinoma. (**c**) grade 2 serous papillary adenocarcinoma. (**d**) Grade 2–3 serous papillary adenocarcinoma. (**e**) Grade 3 serous papillary adenocarcinoma. (**f**) normal ovarian tissue. Microscopy images were taken at 40× magnification. (**D**) Relative RBPMS immunoreactivity staining in the different ovarian cancer types included in the tissue array. (**E**) Western blot of RBPMS protein levels in cisplatin-sensitive and cisplatin-resistant ovarian cancer cells. The whole Western blot image is shown in Supplementary Figure S2.

We took advantage of the Gene Expression Profiling Interactive Analysis (GEPIA) searchable database (RNAseq data) to investigate the RBPMS RNA expression in 426 ovarian cancer patients and 88 normal ovarian patients (controls). We observed that RBPMS levels were significantly lower (* *p* < 0.05) in the ovarian cancer patients when compared with the normal ovarian controls (Figure 1A). KM curves constructed by GEPIA showed that the OS and disease-free survival (DFS) rates were significantly lower in the ovarian cancer patients with lower RBPMS expression levels (Figure 1B). To explore the RBPMS protein expression in the patient tumor tissues, we performed IHC analysis in a high-density tissue array. A total of 69 cases (207 tissue samples) was divided into four ovarian cancer tumor types—serous papillary adenocarcinoma, clear cell adenocarcinoma, mucinous adenocarcinoma, and endometrioid adenocarcinoma—and control groups (normal ovarian and adenocarcinoma tissues).

Supplementary Figure S1 includes a diagram of the composition of the tissue array composition and the distribution within. Table 1 shows the number of tissues per ovarian cancer type, the average age of the patients, and the tumor grade of each of the samples. Figure 1C is a representative image of the RBPMS staining observed in the tissue array of serous papillary adenocarcinoma (non-assigned grade) (Figure 1(Ca)), a grade 1 serous papillary adenocarcinoma (Figure 1(Cb)), a grade 2 serous papillary adenocarcinoma (Figure 1(Cc)), a grade 2–3 serous papillary adenocarcinoma (Figure 1(Cd)), a grade 3 serous papillary adenocarcinoma (Figure 1(Ce)), and a normal ovarian tissue (Figure 1(Cf)). The black arrows in the IHC images indicate positive RBPMS staining. To quantify the RBPMS protein levels in each tissue of the array, the intensity of the staining was categorized as light, medium, or dark. The results showed that the RBPMS immunoreactivity in all the ovarian cancer tissues was weaker when compared with that in the normal ovarian tissues. One hundred percent (138 samples) of the serous papillary adenocarcinoma samples exhibited light-intensity immunoreactivity signals (Figure 1D). In contrast, 100% (27 patients) of the normal ovarian tissues showed dark immunoreactivity to RBPMS. Of the mucinous adenocarcinoma cases, 75% (9 out of 12) of the samples showed medium-intensity immunoreactivity. Of the endometrioid adenocarcinoma tumors, 66% (8 out of 12) of the samples displayed light-intensity immunoreactivity intensity. Of the clear cell carcinoma cases, only 16% (3 out of 18) of the samples exhibited light-intensity immunoreactivity (Figure 1D). Supplementary Table S1 shows the percentage of patients with light, medium, and dark RBPMS staining in each ovarian cancer tumor type and in the normal ovaries.

As we observed that RBPMS was reduced at the RNA and protein levels in serous ovarian cancer patients when compared with normal ovaries, we investigated the RBPMS protein levels in cisplatin-sensitive serous ovarian cancer cells. In a Western blot, we observed that the RBPMS levels were dramatically lower in the cisplatin-resistant compared with the cisplatin-sensitive ovarian cancer cells (Figure 1E). A2780 cells are classified as clear cell carcinomas, while OVCAR3 cells are HGSOCs [24,25]. The half maximal inhibitory concentration (IC50) values of these cells to cisplatin have been published [26,27,28]. Particularly, OVCAR3CIS are 4.5 times more resistant to cisplatin in comparison to their cisplatin-sensitive counterparts, OVCAR3 cells [28].

### 2.2. RBPMS CRISPR/Cas9 Knockout in OVCAR3 Cells

As HGSOC is the most aggressive and mortal gynecological malignancy, we studied the biological and molecular consequences of RBPMS knockout in the HGSOC cell line, OVCAR3. RBPMS knockout was achieved using the CRISPR/CAs9 system and two single-guide RNAs (we called these two RNA guides as SG1 and SG2, respectively) targeting different sequences on exon 2 of the RBPMS gene. We cloned each of the two single guide RNAs individually, in the pSpCas9(BB)-2A-Puro (PX459) V2.0 plasmid and then stably transfected them in the OVCAR3 cells. Additionally, another group of OVCAR3 cells was transfected with an empty vector (EV) CRISPR construct as a control.

**Figure 2:**
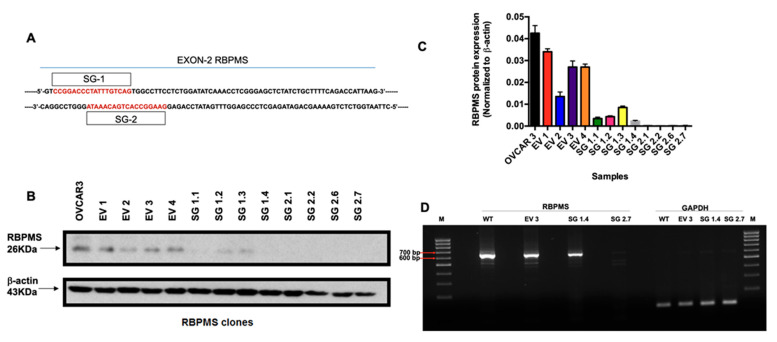
**CRISPR-mediated RBPMS clone generation and validation:** (**A**) RBPMS gene segment showing the sites targeted by the single guide 1 and single guide 2 (SG1 and SG2) RNAs. (**B**,**C**) Western blot and densitometric analysis of the intensity bands, normalized to OVCAR 3. (**D**) RT-PCR of SG1.4 and SG2.7 clones. GAPDH used as a loading control.

Figure 2A shows the portion of the RBPMS gene and the DNA sites targeted by the SG1 and SG2 guide RNAs. Bioinformatics analysis using the Breaking-Cas software that the two selected RNA guides exhibited the lowest off-target effects compared with the other RNA guides generated by the software (data not shown). We obtained and independently grew individual EV, SG1, and SG2 clones. Figure 2B shows the RBPMS protein levels in these clones. Compared with the EV clones, RBPMS protein levels were absent in the SG2 clones; meanwhile, the SG1 clones showed reduced RBPMS protein levels when compared to the EV clones (Figure 2C). We also confirmed the deletion of RBPMS via RT-PCR in the EV, SG1, and SG2 clones. Figure 2D shows that, compared with EV clones, there was no mRNA in the SG2 clones. Moreover, the mRNA levels in the SG1 clones were reduced compared with those in the EV clones. Whole Western blot and gel electrophoresis images are shown in Supplementary Figure S3.

### 2.3. RBPMS Knockout Increased the Proliferation and Invasion Ability of Ovarian Cancer Cells

**Figure 3:**
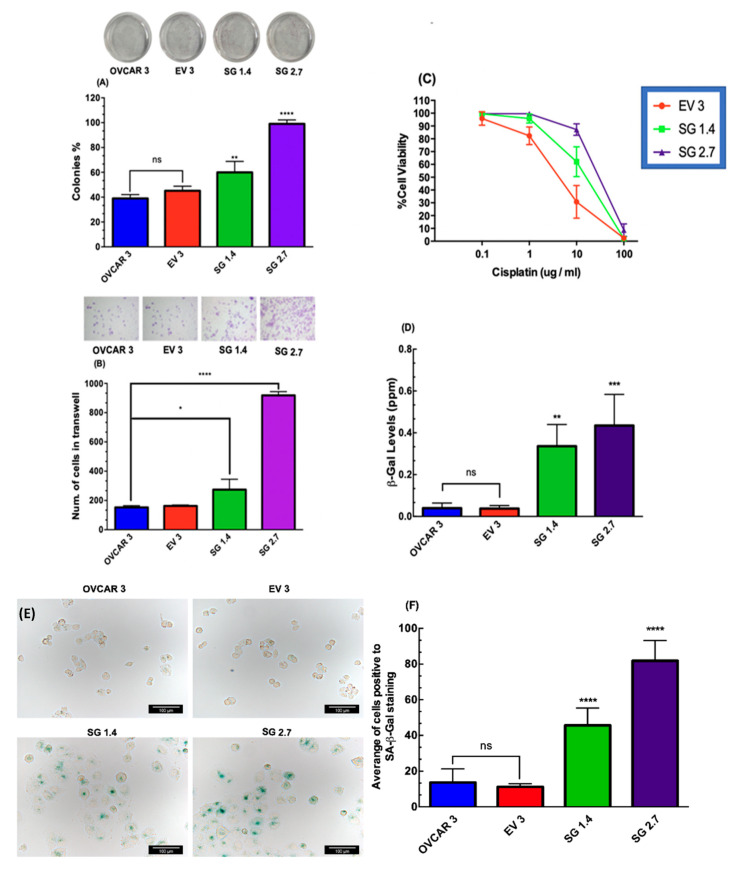
**Effect of RBPMS knockdown on cell growth, proliferation, invasion capacity, and senescence:** (**A**) Colony formation assay. Percentages of clonogenicity were calculated relative to EV cells. (**B**) Cell invasion assay. Percentages of invasion were calculated relative to EV cells. (**C**) EV and SG1 and SG2 clones (3 × 10^4^ cell/mL) were exposed to different concentrations of cisplatin for 72 h. Cell viability values were calculated relative to EV cells. Averages ± SEM are shown for three independent experiments. (**D**) Cells (1 × 10^4^ cells/mL) were plated in Petri diches. Twenty-four hours later, cells were rinsed with PBS, and protein extracts were prepared and diluted at a 1 µg/mL protein concentration. Senescence-associated β-galactosidase activity (SA-β-gal) was assessed via fluorescence. β-galactosidase levels were calculated relative to empty vector cells. Averages ± SEM are shown for three independent experiments. (**E**,**F**) Representative images and quantification of SA-β-Gal-stained cells. Images scale bar: 100 µm (bars: six microscopic fields per condition). * *p* < 0.05, ** *p* < 0.01, *** *p* < 0.001, **** *p* < 0.0001.

We observed that the RBPMS protein levels were lower in the ovarian cancer ovaries compared with the normal ovaries (Figure 1D). Moreover, reduced levels of RBPMS have been associated with accelerated cell proliferation in breast cancer cells [21]. Based on these observations, we investigated whether RBPMS knockout increased the proliferation and invasion ability of OVCAR3 cells. In a clonogenic assay, we observed that the cell proliferation of SG1 and SG2 was significantly higher than that of OVCAR3 untransfected cells or EV clones (** *p* < 0.01 and **** *p* < 0.0001, respectively) (Figure 3A). Particularly, the growth rate of the SG2 clones was more than twice the growth rate of the EV clones (Figure 3A). Similarly, RBPMS knockout increased the invasion ability of the OVCAR3 cells in both SG1 (*** *p* < 0.001) and SG2 (**** *p* < 0.0001) when compared with the EV clone (Figure 3B). Remarkably, the number of invaded cells in the SG2 clones was five times higher than that of the EV clones (Figure 2B).

### 2.4. RBPMS Knockout Reduced the Sensitivity of Ovarian Cancer Cells to Cisplatin Treatment

Based on the observed reduced expression of RBPMS in the cisplatin-resistant ovarian cancer cells (Figure 1E), we next aimed to determine whether RBPMS knockout reduced the sensitivity of ovarian cancer cells to cisplatin treatment. In Figure 3C, we can observe that the SG1.4 and SG2.7 clones were more resistant to cisplatin treatment when compared with the EV clones (IC50s: 18.02, 13.62, and 5.063, respectively). Together, these results suggested that reduced levels of RBPMS increased the cell proliferation and invasion ability and reduced the sensitivity of the ovarian cancer cells to cisplatin treatment.

### 2.5. RBPMS Knockout Increased the Senescence-Associated β-Galactosidase Levels in Ovarian Cancer Cells

Evidence indicates that the acquisition of drug resistance is accompanied by the transformation of cancer cells to a senescence phenotype [28,29]. Thus, we measured the senescence-associated beta-galactosidase (SA-β-Gal) levels in the EV, SG1, and SG2 clones. Figure 3D shows that the β-Gal levels were significantly higher in the SG1 (** *p* < 0.01) and SG2 (*** *p* < 0.001) clones than in the EV clones. To confirm the changes in the β-Gal levels, we obtained images of β-Gal-stained cells. A greater number of SA-β-Gal positive cells were observed in the SG1.4 and SG2.7 clones when compared with the OVCAR3 cells or EV clones (Figure 3E). Figure 3F shows the quantification of the number of SA-β-Gal-positive cells, confirming our observation that reduced levels of RBPMS increased the SA-β-Gal in the ovarian cancer cells.

### 2.6. RBPMS Knockout Altered the Expression of Long-Noncoding RNAs and Protein-Coding Genes Associated with Alteration of the Tumor Microenvironment

**Figure 4:**
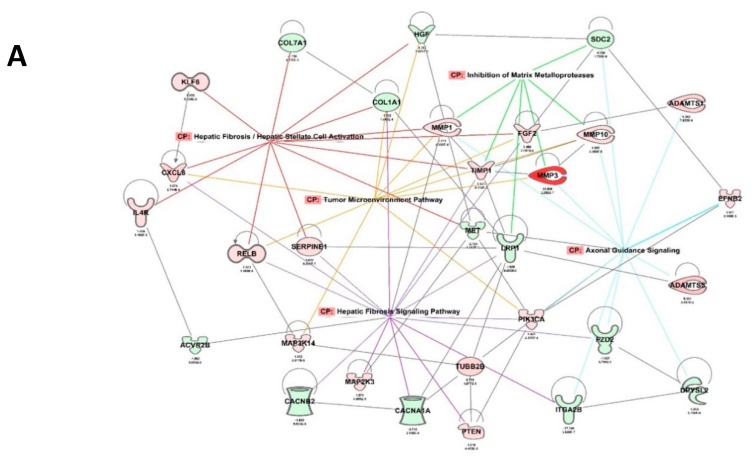

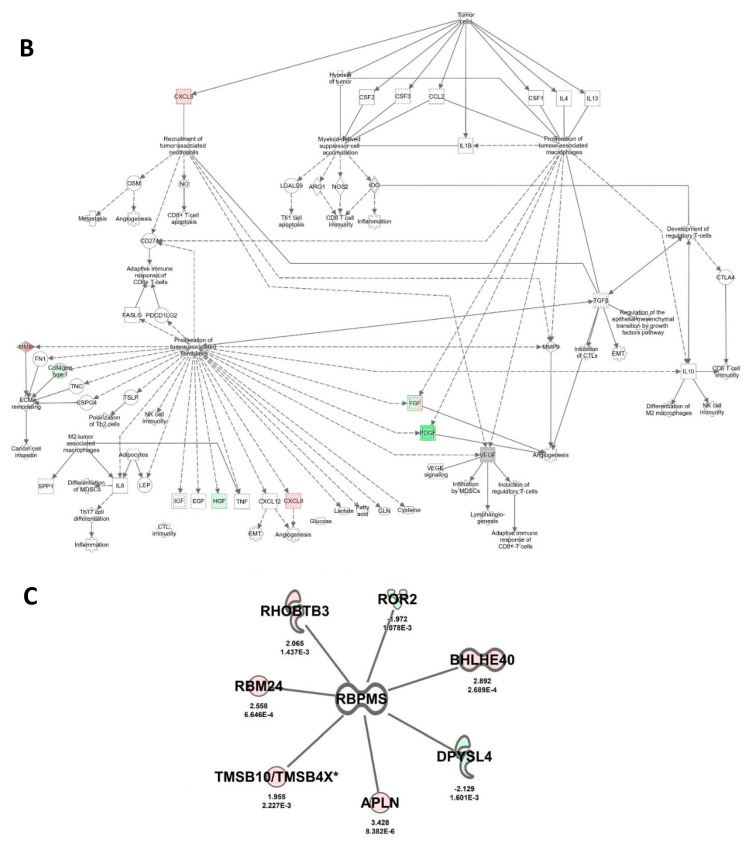
**Ingenuity pathway analysis (IPA) of deregulated transcripts in SG2 and EV clones:** (**A**) Interactions between the top five canonical pathways as found in the RNAseq studies. (**B**) A segment of the tumor microenvironment pathway. (**C**) A node showing seven of the identified transcripts directly interacting with RBPMS. Red color denotes upregulated RNA transcripts, and green denotes downregulated RNA transcripts following RBPMS knockdown.

To further identify changes in the RNA transcripts downstream of RBPMS that can account for the observed biological effects, we performed RNAseq experiments. As the most dramatic effects of RBPMS knockout were observed with the SG2, RNAseq was run in the EV and SG2 clones. We initially identified 1172 RNA transcripts differentially expressed in the EV and SG2 samples (590 up-regulated and 582 downregulated) (Supplementary Table S2). Principal component analysis (PCA) clustered the RNA transcripts of EV and SG2 in two separate regions of the graph (Supplementary Figure S4). Further filtering using a cut-off *p*-value < 0.05 reduced the list of RNA transcripts to 655 (314 upregulated and 341 downregulated in the SG2 and EV clones) (Supplementary Table S3). To better examine the interaction networks of RBPMS downstream genes, lists of differentially abundant genes were uploaded to Qiagen Ingenuity Pathway Analysis (IPA) [30] software for CORE analysis and molecular annotation using the Ingenuity Pathway Knowledge Base. We performed IPA analysis with the 655 differentially expressed transcripts. This analysis produced a list of 98 altered canonical pathways (*p* < 0.05 and fold change ≥ 1.5) (Supplementary Table S4). The top five networks in terms of the number of genes per pathway are depicted in Table 2. These pathways included hepatic fibrosis/hepatic stellate cell activation (21 genes), axonal guidance signaling (31 genes), hepatic fibrosis signaling (26 genes), inhibition of matrix metalloprotease (seven genes), and tumor microenvironment (15 genes) pathways. Figure 4A represents the interaction network of the top five canonical pathways identified in the RNAseq experiments. In this figure, we can observe how some of the downstream-RBPMS effector genes contribute to more than one pathway. For example, collagen type I alpha 1 chain (COL1A1) (downregulated), and the C-X-C motif chemokine ligand 8 (CXCL8) (upregulated) are associated with both the Hepatic Fibrosis/Hepatic Stellate Cell Activation and Tumor Microenvironment pathways. Similarly, the tissue inhibitor of metallopeptidase 1 (TIMP1) is associated with four out of the five pathways: the hepatic fibrosis/hepatic stellate cell activation, hepatic fibrosis signaling, inhibition of matrix metalloprotease, and tumor microenvironment pathways (Figure 4A).

Figure 4B shows the tumor microenvironment network, including the interactions between the cancer cells, cytokine environment, extracellular matrix, immune cell subsets, and other components. The additional top five canonical pathways are included in Supplementary Figure S5. We also generated a cluster that included identified genes directly interacting with RBPMS. As shown in Figure 4C the cluster included seven nodes (the interaction of each gene is not shown to simplify the figure). The genes of this cluster included receptor tyrosine kinase-like orphan receptor 2 (ROR2), RNA binding motif protein 24 (RBM24), apelin (APLN), dihydropyrimidinase-like 4 (DPYSL4), thymosin beta 10 (TMSB10), basic helix-loop-helix family member e40 (BHLHE40), and rho-related BTB domain containing 3 (RHOBTB3). To prioritize the most relevant downstream-RBPMS effectors, we generated a table with the top 20 (10 upregulated and 10 downregulated) differentially regulated RNA transcripts downstream of RBPMS (Table 3). Interestingly, five ncRNAs (lncRNAs and pseudogenes) appeared in this table. An Internet search revealed the biological roles of these genes, which are summarized in Table 4. The deregulation of most of these genes has already been associated with cancer progression (see references in Table 4) [31,32,33,34,35,36]. For example, matrix metalloproteinase 3 (MMP3), one of the most increased transcripts upon RBPMS knockout, has been linked with the metastatic potential of various cancer types [37] as well as cisplatin resistance in ovarian cancer cells [38].

### 2.7. RBPMS Knockout Altered the Levels of Proteins Associated with Cell Integrity, Cell Detoxification, and RNA Processing

We next performed quantitative proteomics experiments to identify changes at the protein level following RBPMS knockout. As the strongest biological effects were observed with the EV and SG2 clones, we ran the proteomics studies for these two groups. OVCAR3 cells were used as an Internet control for the proteomics experiments. Initially, we identified 264 proteins differentially abundant in the SG2 and EV clones (fold change ≥ |1.5|; *p*-value ≤ 0.05) (Supplementary Table S5). After subtraction of the proteins identified in the OVCAR3 and EV clones, we generated a list of 110 proteins (63 increased and 47 reduced) differentially abundant in the SG2 and EV clones (Supplementary Table S6). The lists of these differentially abundant proteins were uploaded to Qiagen IPA software for CORE analysis and molecular annotation using the Ingenuity Pathway Knowledge Base. This analysis produced a list of 42 altered canonical pathways (*p* < 0.05 and fold change ≥ 1.5) (Supplementary Table S7). The top five canonical networks included the LPS/IL-1 mediated inhibition of RXR function (seven proteins), pyrimidine deoxyribonucleotide de novo biosynthesis (three proteins), glutathione-mediated detoxification (three proteins), xenobiotic metabolism PXR signaling (six proteins), and PXR/RXR activation protein pathways (Table 5).

**Figure 5:**
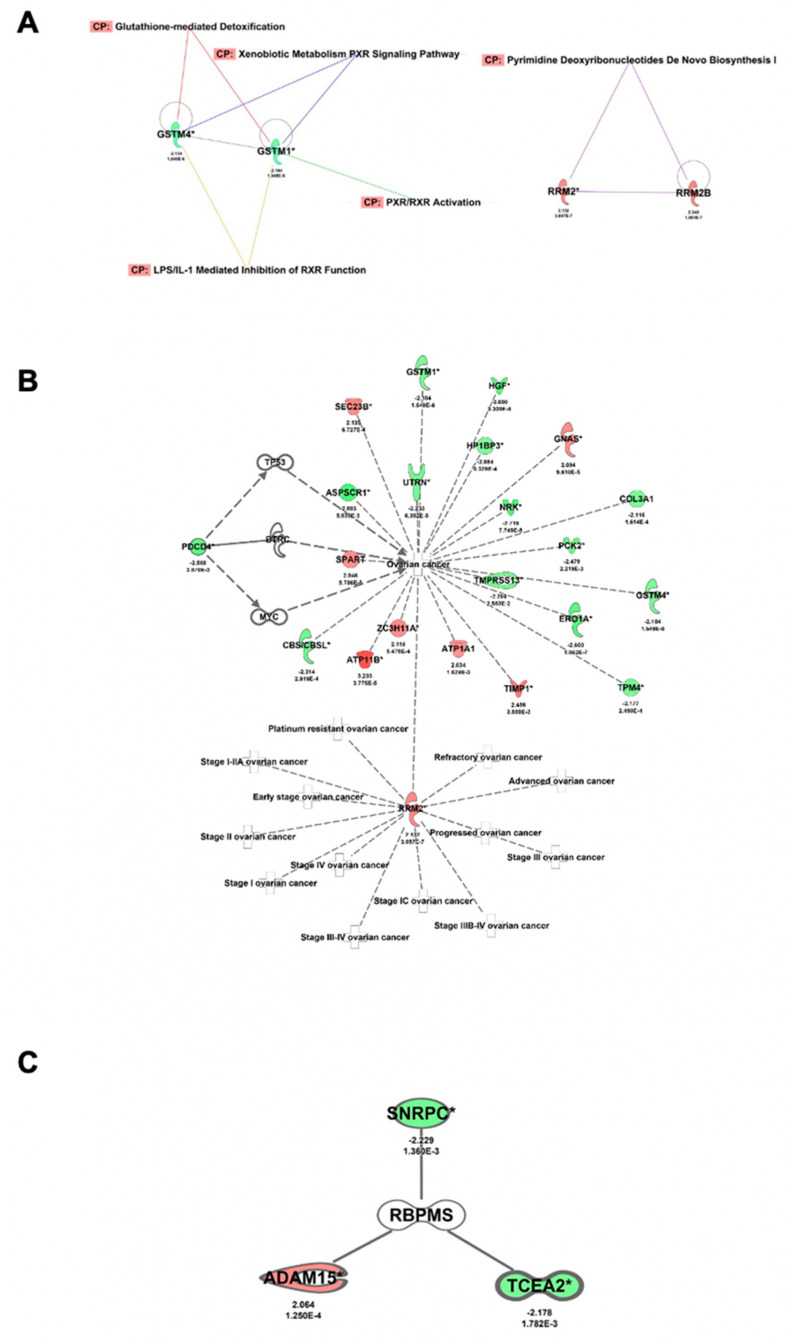
**IPA of differentially abundant proteins in SG2 and EV clones:** (**A**) Interactions between the top five canonical pathways of the proteomic studies. (**B**) Signaling pathway showing proteins associated with ovarian cancer. (**C**) A node showing three of the identified proteins directly interacting with RBPMS. Red color denotes increased protein levels; green color denotes reduced protein levels following RBPMS knockdown.

Figure 5A shows the interaction between these networks. Here, glutathione S-transferase mu 1 (GSTM1) and glutathione S-transferase mu 4 (GSTM4) proteins (reduced in the SG2 vs. EV clones) are nodes of three different networks. Curiously, proteins of the pyrimidine deoxyribonucleotide de novo biosynthesis pathway were unrelated to the other four protein networks (Figure 5A). Each of the top five protein networks is included in Supplementary Figure S6. Using IPA, we also generated a network specific to the ovarian cancer-associated proteins. This network included 23 of the proteins (eight increased and 15 reduced) identified in the proteomics studies (Figure 5B). Of particular interest was ribonucleotide reductase regulatory subunit M2 (RRM2), the increase of which has been associated with all stages of ovarian cancer progression [43]. Programmed cell death 4 (PCDC4), a tumor suppressor gene reduced in many cancer types—including ovarian cancer [51,52]—was also reduced upon RBPMS knockout (Figure 5B). ATP11, which was increased upon RBPMS knockout here (Figure 5B), has also been associated with cisplatin resistance in ovarian cancer [53]. Again, to prioritize the most relevant RBPMS downstream effectors, we generated a table with the top 20 differentially abundant (10 upregulated and 10 downregulated) proteins downstream of RBPMS (Table 6). Further literature searches revealed that the deregulation of many of these proteins plays a role in cell detoxification, cytoskeleton and cell integrity, and RNA processing. This information is summarized in Table 7.

**Figure 6:**
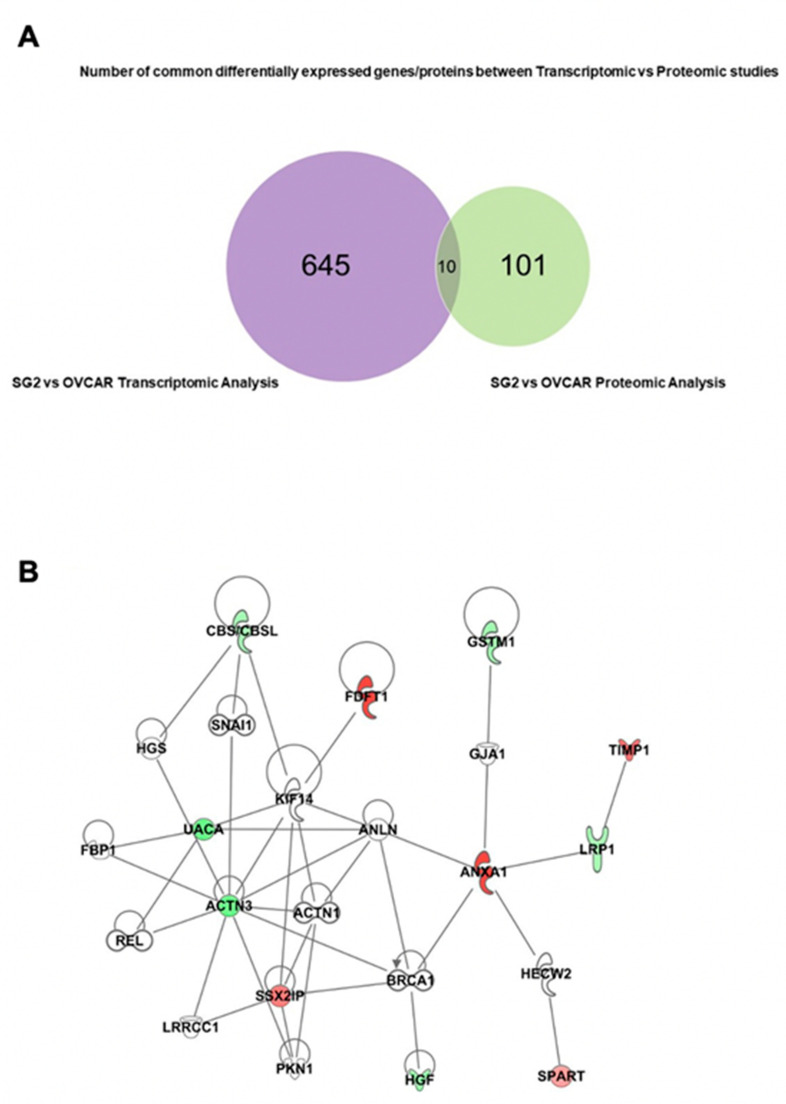
**Common RNA transcripts and proteins deregulated following RBPMS knockout:** (**A**) Venn diagram showing that 655 RNA transcripts and 111 proteins were differentially abundant in SG2 and EV clones. Only 10 genes were common at the RNA and protein levels in the RNAseq and proteomic studies. (**B**) A canonical pathway showing the interaction between the 10 common genes identified via RNAseq and proteomics studies. Red color denotes upregulated and green denotes downregulated genes.

To further determine if the same significantly regulated RNA transcripts were also regulated at the protein level, we compared the list of the 655 RNA transcripts with that of the 110 proteins significantly regulated following RBPMS knockout. The Venn diagram in Figure 6A reveals that only 10 mRNA transcripts were also regulated at the protein level. The differential RNA and protein levels are summarized in Table 8. IPA revealed a strong interaction between these 10 genes, with annexin A1 (ANX1), actin alpha 3 (ACT3), and SSX family member 2 interacting protein (SSX2IP) working as central nodes that interact with the other proteins in the network (Figure 6B).

### 2.8. Prognostic Value of RBPMS Downstream Transcripts

**Figure 7:**
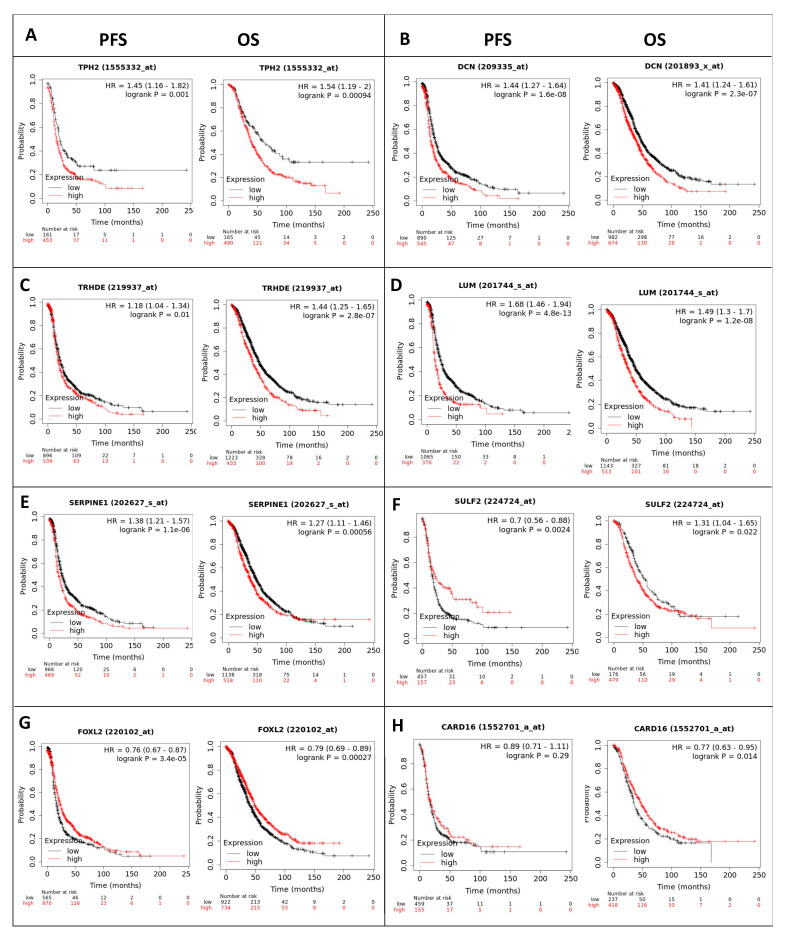
**KM plots:** Survival plots of ovarian cancer patients were generated using the KM plotter database for the top 10 differentially abundant RNA transcripts of the RNAseq experiments-TPH2 (**A**), DCN (**B**), TRHD2 (**C**), LUM (**D**), SERPINE1 (**E**), SULF2 (**F**), FOXL2 (**G**) and CARD16 (**H**). The OS and PFS of the ovarian cancer patients were stratified based on the median RNA expression levels for each gene.

To assess if the differentially expressed RNA transcripts identified upon RBPMS knockout are clinically relevant in ovarian cancer, we conducted a survival analysis using the KM plotter [55]. We interrogated the 20 top genes of the RNAseq experiments (Table 3) (with five ncRNAs not available in the database). According to the median value of the data set, eight RNA transcripts were found to be significantly associated (* *p* < 0.05) with the PFS and OS (Figure 7). Following RBPMS knockout, the expression levels of TPH2, DCN, TRHD2, LUM, and SERPINE1 were in agreement with the survival outcomes: The PFS and OS were worse in ovarian cancer patients with higher levels of these transcripts (Figure 7A–E).

**Figure 8:**
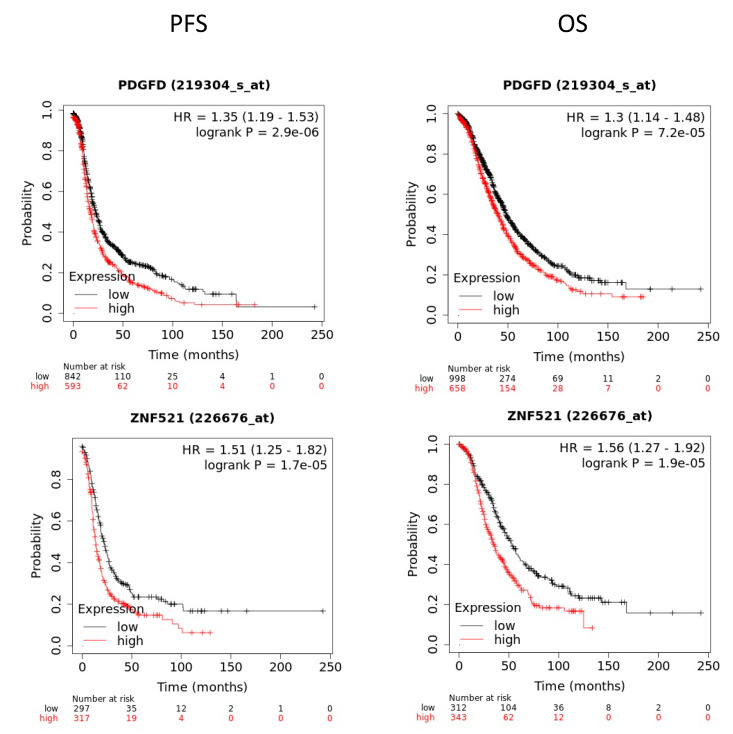
**Survival analysis for PDGFD and ZNF521:** The OS and PFS are lower for ovarian cancer patients with higher levels of these transcripts when compared with ovarian cancer patients with lower levels of these transcripts. However, this tendency does not correlate with our RNAseq results, because the two genes were reduced following RBPMS knockout, and, therefore, we expected an opposite tendency in the KM plotter.

**Figure 9:**
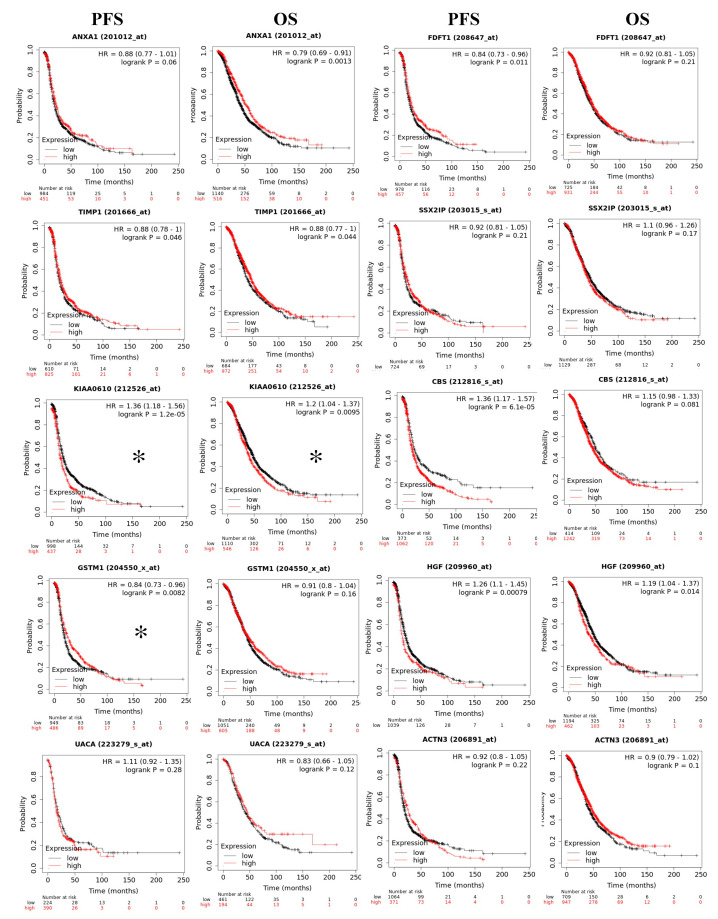
**Survival analysis for the 10 common genes deregulated at the RNAseq and protein levels.** We found a significant correlation (* *p* < 0.05) between the OS and PFS and the RNA levels of KIAA0610 (spartin). We also found a significant correlation between the OS (but not the PFS) and the RNA levels for the GSTM1 gene. These correlations were in agreement with our RNAseq results.

The expression levels of FOXL2 and CARD16 were also in agreement with the survival outcomes: The PFS and OS were better in ovarian cancer patients with higher levels of these transcripts (Figure 7G,H). While the PFS of SULF2 correlated well with its expression levels in the patients, the OS showed an opposite tendency (Figure 7F). Moreover, the PDGFD and ZNF521 levels were significantly associated with the OS and PFS (Figure 8). However, this tendency was opposite to that expected, as these transcripts were reduced after RBPMS knockout. We also interrogated the 10 common genes identified by the RNAseq and proteomics (Figure 6A, Table 7) and found that only the spartin (SPART) (KIAA0610) transcripts levels were significantly associated (* *p* < 0.05) with the PFS and OS of the ovarian cancer patients (Figure 9). Moreover, the transcript levels of GSTM1 were found to be significantly correlated (* *p* < 0.05) with the PFS but not with the OS of the ovarian cancer patients (Figure 9).

## 3. Discussion

The major finding of this study was that the CRISPR-mediated RBPMS knockdown promoted cell proliferation and invasion as well as increased the cisplatin resistance of ovarian cancer cells. We also found that the RBPMS expression levels were reduced in ovarian cancer patients and in cisplatin-resistant ovarian cancer cells when compared with normal ovaries and cisplatin-sensitive cells, respectively. Nakagaki-Silva and co-workers showed that RBPMS is a critical splicing regulator of differentiated vascular smooth muscle cells [19]. Fu et al. reported that RBPMS inhibited the growth and migration of breast cancer cells by repressing AP-1 signaling [21]. Additionally, reduced levels of RBPMS have been documented in bladder cancer and multiple myeloma cell lines [56,57]. However, the biological role of RBPMS in ovarian cancer has not been previously studied.

Using the GEPIA cancer patient database, we observed that the RNA levels of RBPMS were lower in ovarian cancer patients as compared with normal ovary samples. This database also showed that the RNA levels of RBPMS correlated well with the OS of the disease, with ovarian cancer patients with higher RBPMS RNA expression levels living longer than those with lower RBSPMS levels. The RBPMS levels also correlated with the response to therapy, with ovarian cancer patients with higher levels of RBPMS having greater PFS than those with lower RBPMS levels. IHC studies also showed that the immunoreactivity staining of RBPMS in serous ovarian tissues was lower when compared with normal ovaries. Together, this information suggests that RBPMS could represent a novel diagnostic, prognostic, and response-to-therapy marker in ovarian cancer. In the future, we recommend a high number of ovarian cancer samples for IHC studies in particular to confirm our findings.

Our finding that the RBPMS protein levels in cisplatin-resistant ovarian cancer cells were considerably lower than in their sensitive counterparts suggested that this protein could play a key role in cisplatin resistance. To address this hypothesis, we used a CRISPR/Cas9 system. This technology allows for the repair (insertion), knockdown, or knockout of genes using a guide RNA and a dual RNA-guided DNA endonuclease enzyme (Cas9). We designed two guide RNAs, both of which targeted exon 2 of the RBPMS gene. According to the Western blot and RT-PCR experiments, it was observed that the SG2 generated biallelic mutations while guide 1 generated monoallelic mutations, as some RBPMS protein levels were still detected by the Western blots and a band in the gel electrophoresis of the RT-PCR [58,59]. This occurrence of biallelic or monoallelic mutations in OVCAR3 clones should be validated in the future with additional experiments. Nevertheless, the biological effects observed with the guide 2 clones were more dramatic than those observed with the guide 1 clones; this supports our hypothesis regarding biallelic vs. monoallelic mutations.

The CRISPR-mediated RBPMS knockdown of the protein levels in the ovarian cancer cells induced long-term effects in terms of cell proliferation, as evidenced by the clonogenic assays. RBPMS knockdown also reduced the invasiveness of the OVCAR3 cells. Our findings agree with the report of Fu et al., who argued that RBPMS inhibited the proliferation and migration of breast cancer cells by blocking the formation of c-Jun/c-Fos or c-Jun/SMAD3 complexes [21]. We also observed that the knockdown of RBPMS reduced the sensitivity of the ovarian cancer cells to cisplatin treatment. The idea that RBPMS could be involved in the drug resistance of cancer cells originated in the studies of Rastgoo et al., who showed that increased levels of the enhancer of zeste homolog 2 (EZH2), the catalytic subunit of polycomb complex (PRC2), contributes to drug resistance in multiple myeloma (MM) by downregulating RBPMS [56]. In the same study, the restoration of RBPMS by miR-138 overexpression re-sensitized the MM cells to bortezomib (BTZ), a proteasome inhibitor that is FDA-approved against MM [56].

Our results indicated that the decreased expression of RBPMS promotes cell proliferation, invasion, and drug resistance. Fu et al. reported that, in breast cancer cells, specific RBPMS isoforms bind to c-Fos/s-Jun and/or c-Jun/SMAD3-4, repressing the expression of genes regulated by the AP-1 complex (including c-Jun, c-Fos, and other proteins) [21]. The increased expression of c-Jun and c-Fos has been associated with cell proliferation and drug resistance in ovarian cancer [60,61,62]. However, the molecular effectors downstream of RBPMS in ovarian cancer cells have not been studied. We took advantage of our CRISPR-mediated RBPMS-knockdown clones to perform RNAseq, observing changes in the expression of several RNA transcripts, including lncRNAs and genes associated with the tumor microenvironment. For example, decorin (DCN), a tumor suppressor gene (upregulated in RBPMS knockdown clones) affects the biology of various types of cancers by targeting a number of crucial signaling molecules involved in cell growth, survival, metastasis, and angiogenesis [63,64]. According to such reports, we expected a reduction in the DCN levels upon RBPMS knockout. The way in which increased levels of DCN are associated with cisplatin resistance needs further clarification. Moreover, the upregulation of matrix metallopeptidase 3 (MMP3) has been associated with cancer metastasis and tumor growth in breast cancer [65]. In fact, public microarray data from our laboratory (Gene Expression Omnibus accession) has shown that cisplatin-resistant ovarian cancer cells in the A2780CP20 cell line expressed higher MMP3 levels than their cisplatin-sensitive counterparts of the A2780 cell line. Furthermore, we recently published that MMP3 is post-transcriptionally regulated by miR-18a-5p, which is decreased in cisplatin cells [38]. Aldehyde dehydrogenase 1A1 (ALDH1A1) is associated with the capacities of self-renewal, differentiation, and tumor initiation [66]. High levels of ALDH1A have been found to promote invasion, metastasis, and poor outcomes in human esophageal squamous cell carcinoma [66]. Together, this information suggests that RBPMS regulates the expression of genes via the self-renewal capacity as well as invasion and metastasis in ovarian cancer cells. Additionally, SERPINE1 has been proposed as an oncogene that promotes drug resistance in breast cancer cells [67,68]. The upregulation of FOXL2 (downregulated upon RBPMS knockout) has been found to suppress cervical cancer cell proliferation and facilitate the apoptosis of these cells [69,70]; thus, it could be important to study the role of FOXL2 in ovarian cancer. Furthermore, the deregulation of other downregulated genes following RBPMS knockout, including PDGFD, TES, CARD16, and KNF521, has been linked to the progression of many cancer types [71,72,73], and the role of these genes in the ovarian cancer setting should be investigated.

Of the top 10 RBPMS regulated transcripts, we identified five lncRNAs. LncRNAs have garnered increasing interest over the past years, as these molecules regulate cell function at the DNA, RNA, and protein levels and are commonly dysregulated in cancer [74]. TRHDE-AS1, the top upregulated gene following RBPMS knockout, is an antisense ncRNA downregulated in lung cancer [75,76]. PURPL, a lncRNA downregulated in RBPMS knockout clones, regulates p53 unction in many cancer types [77]. As p53 gain-of-function (GOF) mutants have been reported in many cancer types [78,79,80], it could be interesting to investigate if increasing PURPL levels could reduce the p53 levels in cisplatin-resistant ovarian cancer cells. The role of the other three deregulated ncRNAs upon RBPMS knockout—LINC01036 (upregulated), LOC101927789 (downregulated), and LOC105377329 (downregulated)—have not yet been studied in cancer.

Importantly, when we investigated the potential clinical significance of each of the top 20 deregulated RNA transcripts using the KM plotter database, the expression of eight of the 20 genes correlated well with the OS and PFS of the disease. Ovarian cancer patients with higher RNA levels of TPH2, DCN, TRHDE, and LUM have lower OS when compared with ovarian cancer patients with lower levels of these genes. In contrast, ovarian cancer patients with high RNA levels of SERPINE1, SULF2, FOXL2, and CARD16 live longer in comparison with ovarian cancer patients with lower levels of these RNA transcripts. The PFS, which is indicative of the response to therapy, followed the same tendency for the eight RNA transcripts. These results suggested that these eight genes together with RBPMS could be used as prognostic and/or as response-to-therapy biomarkers in ovarian cancer. Additional patient databases and prospective studies should be respectively reviewed and conducted to confirm these results.

Among the increased proteins following RBPMS knockdown, the two ARRB members—ARRB1 and ARRB2—are deregulated in many cancer types and have been linked to the inhibition of the senescence and growth as well as the promotion of the self-renewal, progression, and invasion of cancerous cells [39,40,81]. Moreover, CPT1A plays a crucial role in radioresistant and chemoresistant phenotypes in diverse cancer types [41,42,82]. Equally, altered protein levels of FLNA, HSPA7, HGF, ERO1A, and GARS1, have been related to cancer migration, adhesion, poor patient prognosis, and metastasis [48,49,50,83,84]. Particularly, filamin A (FLNA), a cytoskeleton regulator, has been reported as taking on opposite roles depending on the cancer type [47]. Both GSTM1 and GSTM2 play important roles in cell detoxification, protecting cells against cancer [45,54]. Thus, it may be interesting to study whether increased levels of these proteins will reduce the sensitivity of ovarian cancer cells to cisplatin treatment. The consequences of the reduced expression of PABPC4L (also reduced in RBPMS knockout clones) has not yet been studied in cancer; however, this protein plays a critical role during protein synthesis, as it travels together with the mRNA transcripts from the nucleus to the cytoplasm and promotes eIF4F assembly in the ribosomes for translation initiation [44,85].

Curiously, only 10 differentially abundant RNA transcripts were also detected at the protein level: ANXA1, FDFT1, TIMP1, SSX2IP, SPART, CBS/CBSL, GSTM1, HGF, UACA, and ACTN3. Importantly, all of these genes showed the same expression tendency at the RNA and protein levels. In breast cancer cells, high levels of ANXA1 have been linked with changes in the tumor microenvironment via altering the inflammatory response of tumor-associated macrophages [86]. Moreover, high levels of farnesyl-diphosphate farnesyltransferase 1 (FDFT1) have been implicated in the development of certain types of cancers [87,88]. However, in other cancer types, this protein acts as a tumor suppressor gene [89]. Interestingly, in cisplatin-resistant ovarian cancer cells, the expression of FDFT1 was found to be increased by sterol regulatory element binding transcription factor 2 (SREBP2) [90]. Accordingly, it could be important to study the role of RBPMS and SREBP2 in the context of cisplatin resistance [91]. On other hand, high levels of tissue inhibitor matrix metalloproteinase-1 (TIMP1) have been associated with poor clinical outcomes in several cancer types [92,93]. Moreover, the overexpression of SSX family member 2 interacting protein (SSX2IP) has been documented in patients with acute myeloid leukemia (AML) [94] and found to promote metastasis and chemotherapeutic resistance in hepatocellular carcinoma cells [95].

We also observed the reduced expression of cystathionine β-synthase (CBS), an enzyme that regulates homocysteine metabolism and hydrogen sulfide (H_2_S) biosynthesis. Homocysteine and H_2_S control cellular energetics, redox status, DNA methylation, and protein modification [96]. Bhattacharyya et al. showed that high levels of CBS promote ovarian cancer progression and drug resistance [97]. Additional studies should be performed to clarify the role of CBS in the cisplatin resistance of ovarian cancer. The reduced expression of GSTM1 has been associated with certain cancer types [46]. However, the way in which the reduced expression of this gene correlates with cisplatin resistance is currently unknown. Hepatocyte growth factor (HGF) and its receptor, Met, play key roles in cell motility, with increased levels of HGF/Met having been associated with cell migration, invasion, and drug resistance in several cancer cells [98]. Moreover, the uveal autoantigen with coiled-coil domains and ankyrin repeats (UACA/Nucling) is upregulated in various cancers types [99,100]. Again, however, the way in which the reduction in HGF and UACA correlate with the cisplatin resistance of ovarian cancer needs further investigation. Finally, the role of actinin alpha 3 (ACTN3), a protein involved in crosslinking actin-containing thin filaments [84], has not been studied in cancer. In this study, we focused our attention on the top 20 RNA transcripts and top 20 proteins downstream of RBPMS. However, 655 mRNA transcripts and 110 proteins were differentially abundant between the RBPMS knockout clones and the controls. The biological and clinical significance of many of these genes should be investigated in the future. Additionally, the role of many of these genes should be studied in the context of the increased senescence upon RBPMS knockout. Although chemotherapy-induced senescence has short-term benefits for cancer treatment, chemotherapy also causes the reprogramming of gene expression, which leads to the selection of highly drug-resistant phenotype clones during senescence [29]. Therefore, it is important to elucidate which of the RBPMS downstream pathways reported here are responsible for the increasing senescence in cisplatin-resistant ovarian cancer.

In summary, the major finding of this study was that RBPMS knockout increased the proliferation and invasion ability of ovarian cancer cells as well as their senescence. Decreased RBPMS expression also reduced the sensitivity of the ovarian cancer cells to cisplatin treatment. Furthermore, several RBPMS downstream effectors have been identified in this study. Some of them, including RBPMS, could be used as prognostic response-to-treatment biomarkers. The upregulated RBPMS downstream regulated genes could be explored as targets for therapy. As various RBPMS splice variants have been reported, future research should elucidate the specific splice RBPMS isoform responsible for the reported biological effects as well as how this regulation of RBPMS downstream targets occurs. Importantly, Yang et al. recently reported that the low abundance of a circular RBPMS correlated with aggressive phenotypes in bladder cancer [57]. Increased levels of this circular RNA suppressed cell proliferation, invasion, and migration by directly targeting miR-330-3p and retinoic acid induced 2 axis (RAI2) [57]. Therefore, the elucidation of the biological role of the RBPMS and its splice variants represent an important research area in the cancer field.

## 4. Materials and Methods

### 4.1. Cancer Databases Examination

The GEPIA searchable database (http://gepia.cancer-pku.cn/detail.php, accessed on 19 October 2021) was used to assess correlations between the RBPMS expression levels and patient outcomes. This database includes 426 ovarian cancer tumors and 88 normal ovarian tissues. KM survival analysis was performed using publicly available gene chip and RNA-Seq datasets in the KM plotter (www.kmplot.com) [55]. For each gene symbol, a probe ID was selected, and the ovarian cancer patients were categorized into high- and low-expression groups based on the RNA expression median values of the dataset. For genes with multiple probes, the best probe was selected. All available datasets were used for survival analysis. KM survival plots for OS and PFS were generated with their respective hazard ratios (HRs), confidence intervals (CIs), and *p*-values (log-rank). *p*-values < 0.05 were considered statistically significant.

### 4.2. RBPMS Immunohistochemistry in an Ovarian Tissue Array

A high-density epithelial ovarian carcinoma tissue array (http://www.biomax.us/tissue-arrays/Ovary/OV2083, accessed on 30 November 2021) was purchased from US Biomax, Inc. (Rockville, MD, USA). The tissue microarray contained 69 cases/208 cores, with triplicate cores per case (1 mm, 5 μm in each). Briefly, the slides were deparaffinized, re-hydrated, and immersed in distilled water with 3% hydrogen peroxidase to suppress endogenous peroxidase activity. Antigen retrieval was performed via microwave treatment in an antigen unmasking solution (Vector Laboratories, Inc, Burlingame, CA, USA) for 15 min. Sections were incubated with RBPMS antibody (Abcam, Cambridge, MA, USA) at a dilution of 1:100 in the Dako antibody diluent (Dako North America Inc., Carpinteria, CA, USA) overnight at 4 °C. Subsequently, the Envision peroxidase-labeled polymer HRP (goat anti-rabbit ready-to-use form; Dako North America Inc, Carpinteria, CA, USA) was applied to the sections, and signals were developed with diaminobenzidine (DAB) chromogen (Dako North America Inc, Carpinteria, CA, USA). The staining intensities were assigned using a color scale (score 1: negative/weak staining intensity; score 2: intermediate staining intensity; and score 3: strong staining intensity).

### 4.3. Cells and Culture Conditions

The human A2780CP20 ovarian epithelial cancer cells were provided by Dr. Anil K. Sood (MD Anderson Cancer Center, Houston, TX, USA) and have been described elsewhere [12,26,27,28,101,102]. The A2780 and A2780CIS cells were purchased from the European Collection of Cell Cultures (ECACC, Porton Down, Salisbury, UK), and the OVCAR3 cells from the American Type Culture Collection (ATCC, Manassas, VA, USA). The OVCAR3CIS cells were generated by exposing OVCAR3 to increasing concentrations of cisplatin (CIS; Sigma-Aldrich, St. Louis, MO, USA), as previously described [28]. The IC50 values and molecular characterization of these cells (A2780, A2780CP20, A2780CIS, OVCAR3, and OVCAR3CIS) have been previously published [28,38,103]. The cells were maintained in RPMI1640 (GE Healthcare Life Sciences, Logan, UT, USA; A2780, A2780CP20); RPMI1640 + insulin (0.01 mg/mL; Sigma-Aldrich, St. Louis, MO, USA; OVCAR3, OVCAR3CIS) supplemented with 10% fetal bovine serum (FBS; HyClone); and 0.1% antibiotic/antimycotic solution (HyClone) at 37 °C in 5% CO_2_ and 95% air. All cell lines were screened for mycoplasma using the LookOut^®^ Mycoplasma PCR detection kit (Sigma-Aldrich, St. Louis, MO, USA), and authenticated by Promega (Madison, WI, USA) and ATCC using Short Tandem Repeat (STR) analysis. In vitro assays were performed at a 70–85% cell density.

### 4.4. CRISPR Design

We used the Breaking-Cas web server sgRNA design tool (http://bioinfogp.cnb.csic.es/tools/breakingcas, accessed on 20 October 2020) to design two single guide RNAs (SG1 and SG2). The input NCBI Reference Sequence was NG_029534.1 (homo sapiens RNA binding protein, mRNA processing factor (RBPMS); RefSeqGene on chromosome 8) (Ensembl: ENSG00000157110) corresponding to the mRNA sequence NM_001008710.3 (homo sapiens RNA binding protein, mRNA processing factor (RBPMS); transcript variant 1), which encodes for the canonical RBPMS isoform. We selected the two guide RNAs (gRNAs) with lower estimated off-target effects as predicted by the Breaking-Cas program. The sequence of SG1 as 5′-CCGGACCCTATTTGTCAG-3′, which binds to the RBPMS DNA region 95,354–95,371 (621–638 mRNA region), and that of SG2 was 5′-GAAGGCCACTGACAAATA-3′ (-strand), which binds to the RBPMS genomic region 95,379–95,362 (646–629 mRNA region). Both the SG-1 and SG-2 binding regions are located on exon 2 of the RBPMS gene. The forward and reverse primers of each RNA guide were purchased from Sigma. Each pair of oligonucleotides were separately cloned into the pSpCas9(BB)-2A-Puro (PX459) V2.0 plasmid using the golden gate assembly cloning strategy [104,105] (Addgene, plasmid #62988). Sanger sequencing was used to confirm the correct CRISPR cloning into this vector. The OVCAR3 cells (6 × 10^4^ cells/mL) were stably transfected (with lipofectamine 3000, Fisher Scientific, Waltham, MA, USA) with the empty vector or with the RBPMS-SG-1 or CRISPR-RBPMS SG-2 vectors. Individual clones were selected with puromycin.

### 4.5. RNA Isolation, cDNA Synthesis, and PCR

The GenElute™ Mammalian Total RNA Miniprep Kit (Sigma-Aldrich, St. Louis, MO, USA) was used to isolate the total RNA from the CRISPR clones following the manufacturer’s instructions. The RNA purity and quantity were assessed with the spectrophotometer NanoDrop (Fisher Scientific, Waltham, MA, USA). The cDNA was synthesized starting with each RNA sample using the Enhanced Avian RT First-Strand Synthesis Kit (Sigma-Aldrich, St. Louis, MO, USA). Briefly, the total RNA (500 ng), 500 µM dNTP (deoxynucleotide mix), 2.5 µM random primers, and nuclease-free water were mixed to a 10 µL final volume. The mixture was gently and briefly centrifuged and heated at 70 °C for 10 min in a thermal cycler. Next, 2 µL of 10X eAMV-RT buffer, 1 µL of Enhanced Avian RT, 1 µL of RNase inhibitor, and nuclease-free water were added to the reaction, resulting in a final volume of 20 µL. Samples were then incubated at 25 °C for 15 min followed by incubation at 45 °C for 50 min. PCR was performed in a PCR system (Fisher Scientific, Waltham, MA, USA). Each PCR reaction tube contained 25 µL of JumpStart REDTaq ReadyMix (Sigma-Aldrich, St. Louis, MO, USA), 0.3 µM of each forward and reverse primes, 200 ng of the cDNA product, and nuclease-free water, resulting in a final volume of 50 µL. The cycling parameters were: initial denaturation at 94 °C for 2 min, 30–35 cycles; denaturation at 94 °C for 30 s; Annealing at 61 °C for 30 s; extension at 72 °C for 2 min, and final extension at 72 °C for 5 min followed by a hold at 4 °C. Primers: RBPMS forward: 5′-AGGAAGGACCGGGAAGATGA-3′, RBPMS Reverse: 5′-CACAAGACAGATTGCAGCCG-3′, Beta-actin (β-actin) was used as endogenous control, Primers: β-actin forward: 5′-CCCTTTTTGTCCCCCAAC-3′, β-actin reverse: 3′-CTGGTCTCAAGTCAGTGTACAGGT-5′ (annealing temperature of 60 °C). The PCR products were separated via electrophoresis in 1.5% agarose gel. The gel images were obtained in a Gel Doc XR+ instrument (BioRad, Hercules, CA, USA) after gel staining with ethidium bromide.

### 4.6. Western Blot Analysis

Cells were collected and washed a few times with phosphate buffer saline (PBS) and stored at −80 °C until use. Cell pellets were lysed with ice-cold lysis buffer (1% Triton X, 150 mmol/L NaCl, 25 mmol/L Tris HCl, 0.4 mmol/L NaVO_4_, 0.4 mmol/L NaF, and a protease inhibitor cocktail from Sigma), and the total protein concentration was determined using Bio-Rad DC Protein Assay reagents (Bio-Rad, Hercules, CA, USA). The protein samples were separated via SDS-PAGE and blotted onto nitrocellulose membranes. The membranes were blocked in either 5% non-fat dry milk (BioRad, Hercules, CA, USA) or 5% BSA (HyClone) Sigma-Aldrich, St. Louis, MO, USA and probed with primary antibodies (anti-RBPMS, 1:1000 dilution) or anti-β-actin (Sigma, St. Louis, MO, USA; 1:10,000 dilution). The membranes were then incubated with mouse or rabbit IgG horseradish peroxidase (HRP)-linked secondary antibodies (Cell Signaling, 1:5000 dilution) followed by enhanced chemiluminescence and autoradiography. Bands were imaged with a Bio-Rad Gel Doc XR+, and the signal intensity of each band was quantified using Image Lab software (BioRad, Hercules, CA, USA).

### 4.7. Colony Formation and Invasion Assays

For the assessment of cell growth, colony formation assays were conducted using crystal violet dye (Sigma-Aldrich, St. Louis, MO, USA). Briefly, RBPMS CRISPR clones (3 × 10^4^ cells/mL) were seeded into 6-well plates. Twenty-four hours later, 1000 cells were seeded into 10 cm Petri dishes. Ten days later, colonies were fixed and stained with 0.5% crystal violet solution in methanol. Colonies of at least 50 cells were scored in five random fields using a light microscope (CKX41; Olympus, Center Valley, PA, USA) with a total magnification of 40X. Cell invasion was analyzed using the Matrigel transwell method as previously described [15,106]. The cells (3.5 × 10^4^ cells/mL) were seeded into 6-well plates. The next day, Matrigel (BD Biosciences, San Jose, CA, USA) in serum-free medium was added onto the upper chambers of 24-well transwell plates and incubated at 37 °C for polymerization. Clones were collected and resuspended in serum-free medium and re-seeded onto the Matrigel-coated chamber. Medium containing 10% FBS was added to the lower wells. After 48 h at 37 °C, the medium was removed, and cells that had invaded through the Matrigel were fixed and stained using the Protocol Hema 3 Stain Set (Fisher Scientific, Kalamazoo, MI, USA). The invading cells were counted at 20X using an Olympus 1X71 microscope equipped with a digital camera (Olympus DP26). The cell invasion percentages were calculated by assuming the empty values in terms of 100% cell invasion.

### 4.8. Cell Viability Assays

To determine the cell viability of RBPMS, the CRISPR clones (2 × 10^4^ cells/mL) were seeded into 96-well plates. Twenty-four hours later, the cells were exposed to different concentrations of cisplatin (Sigma-Aldrich, St. Louis, MO, USA). Seventy-two hours after cisplatin treatment, the medium was removed, and Alamar blue dye (Invitrogen, Thermo Fisher Scientific, Eugene, OR, USA) was added following the manufacturer’s instructions. The optical density (OD) values were obtained using a plate reader (BioRad, Hercules, CA, USA), and the cell viability percentages were calculated after blank OD subtraction, taking the untreated cell values as 100% of cell viability.

### 4.9. Senescence-Associated β-Galactosidase Activity

Senescence was measured with the beta-galactosidase (β-Gal) Detection Kit from Abcam (catalog #AB176721). The fluorescein di-β-D-galactopyranoside (FDG) substrate kit generates a fluorescent product that can be measured. To summarize, the cells were collected, lysed with protein lysis buffer, and diluted for a final protein concentration of 1 µg/mL. The protein samples were incubated with FDG for four hours. After incubation, a stop buffer was added, and fluorescence was quantified in a Thermo Scientific Varioskan Flash spectral reader machine at 490 nm excitation and 525 nm emission. Following this, the β-Gal levels of each sample were calculated with respect to the β-galactosidase standard curve prepared for each experiment. To assess the senescence associated β-galactosidase staining, we seeded 30,000 of each cell type (OVCAR3, EV, SG1, and SG2 clones) per well in a 6-well plate. After 24 h, the β-galactosidase staining was assessed using a senescence detection kit (Ab65351, Abcam, Cambridge, MA, USA) following the manufacturer’s recommendations. Cell images were taken at 20X on an Olympus 1X71 microscope.

### 4.10. RNA Sequencing Library Preparation, Data Processing, and Statistics

For RNA sequencing library preparation total RNA was extracted from cell pellets using the Qiagen RNeasy Kit (Cat #74004). The RNA quality was checked using Agilent RNA TapeStation, and 1 ug of high-quality RNA was used for polyA mRNA enrichment (RIN > 9.7). The NEBNext polyA mRNA magnetic isolation module (NEB #E7490) was used for purification of the polyA mRNAs according to manufacturer protocol. The isolated mRNA was then fragmented into ~200 bp fragments and further purified for use in library preparation. cDNA preparation and adaptor ligation were performed according to the manufacturer protocol, and the DNA was amplified for eight PCR cycles. The final library was purified using NEBnext sample purification beads, and quality control was performed using Agilent HS-DNA Tapestation analysis. The samples were multiplexed for a final concentration of 5 nM and sequenced on the Novaseq platform. Files containing RNA sequencing reads were adapter and quality-trimmed using TrimGalore-0.6.0. Bowtie2 (version 2.2.9) was used to remove contaminating reads from ribosomal RNA and transfer RNA [107,108,109]. The trimmed and contamination-filtered reads were mapped to the hg38 genome (GENCODE Release 31) using STAR aligner version 2.5.2a, and a count matrix was obtained using the “Gene Counts” option [110]. The DESeq2 (version 1.28.1) package was used to perform a differential expression analysis using R version 4.0.1 [111]. As the data was in two batches, a batch correction term was introduced in the DESeq2 model. The Ensembl IDs were converted to gene symbols and names using the org.Hs.eg.db package (version 3.11.4). Significance was set at an FDR-adjusted *p*-value < 0.05 and log2 fold change > 1.

### 4.11. Proteomics, Data Processing, and Statistics

*Sample Processing*: Whole cell protein lysates of were collected for mass spectroscopy (MS). The protein lysates of OVCAR3 cells were used as an internal control. MS was run in triplicates with three different protein pellets per condition. Aliquots (100 µg) were acetone-precipitated, and pellets were resuspended in sample buffer at 2 µg/μL. The samples were loaded into precast TGX Mini-Protean gels and Coomassie-stained. The protein bands were cut out, and gel pieces were destained via incubation with 50 mM ammonium/50% acetonitrile solution at 37 °C for up to 3 h. Thereafter, the samples were reduced with dithiothreitol (25 mM DTT in 50 mM ammonium bicarbonate) at 55 °C, alkylated with iodoacetamide (10 mM IAA in 50 mM ammonium bicarbonate) at room temperature in the dark, and digested with trypsin (Promega) overnight at 37 °C. The trypsin/protein ratio utilized for optimal digestion was 1:50. The next day, the digested peptides were extracted out of the gel pieces using a mixture of 50% acetonitrile/2.5% formic acid in water. The extracted peptides were dried and stored at −80 °C until TMT labelling. *TMT labeling:* As specified by the manufacturer’s protocol (Fisher Scientific, Waltham, MA, USA), the dried extracted samples were reconstituted in 100 mM triethyl ammonium bicarbonate (TEAB), a dissolution buffer, and labeled with TMT10plex labelling reagents (41 μL, 0.8 mg). The addition of the labeling reagents was followed by one-hour incubation and a quenching step of 15 min. Finally, equal amounts of each sample were mixed to generate a final pool. We only used about 20% of the volume per sample for this pool to avoid overloading the fractionation columns in the next step. The remaining 80% of the sample volume was stored in individual vials at −80 °C and was available to be repeated with new TMT pools if needed. The final pools were dried for fractionation, which will be discussed in the following section. *Fractionation*: This procedure was performed using the Pierce High pH Reversed-Phase Peptide Fractionation Kit (Reference #89875) and following the manufacturer’s instructions. Briefly, the column was conditioned twice using 300 μL acetonitrile and centrifuged at 5000× *g* for 2 min; these steps were repeated using 0.1% trifluoroacetic acid (TFA). Each TMT-labeled pool was reconstituted in 300 μL of 0.1% TFA, loaded onto the column, and washed to remove salt contaminants or any unbound TMT reagent. The clean pooled sample was then eluted eight times into eight different vials using a series of elution solutions with different acetonitrile/0.1% triethylamine ratios. The elution solutions are specified in the manufacturer’s protocol. The entire procedure was performed with two different pools generated per TMT kit, and 16 fractions were recovered. These fractions were then dried prior to LC-MS/MS analysis. *Sample Preparation for LC-MS/MS*: The reconstitution of the dried fractionated peptides for mass spectrometry analysis was made using 0.1% formic acid in water (Buffer A). A small portion was transferred to a special sample vial for the injection of 2 μL of the sample into the Q-Exactive Plus instrument. *LC-MS/MS Analysis*: For peptide separation, a PicoChip H354 REPROSIL-Pur C18-AQ (3 μm, 120 A) (75 μm × 105 mm) chromatographic column was used. The separation was obtained using a gradient of 7–25% of 0.1% of formic acid in acetonitrile (Buffer B) for 102 min, 25–60% of Buffer B for 20 min, and 60–95% of Buffer B for 6 min. This led to a total gradient time of 128 min at a flow rate of 300 nL/min—with an injection volume of 2 μL per sample. A full scan (MS1) was measured for the *m*/*z* range of 375 to 1400 at a resolution of 70,000. The MS2 (MS/MS) analysis was configured to select the 10 most intense ions (“top 10”) for HCD fragmentation at a resolution of 35,000. A dynamic exclusion parameter was set at 30 s. *Database Search*: The raw data files obtained after the mass spectrometry analyses were searched with a human (Homo sapiens) database downloaded from Universal Protein Resource (UniProt) in July of 2020. The raw data was analyzed using Proteome Discoverer software version 2.2. A dynamic modification for oxidation +15.995 Da (M) was configured. A static modification of +57.021 Da (C)—generated by the alkylation during processing—and static modifications from the TMT reagents +229.163 Da (Any N-terminal K) were all included in the parameters for the search. *Results*: The results for the quantitative proteomic analyses given by the database search are given in Excel format. Note that, for each protein, you can access all the peptides identified by the program with their respective labeled reporter ions by clicking the cross icon on the left of the Excel worksheet. *Bioinformatic analysis*: The analysis was performed with the Bioconductor software limma [112,113]. The statistical analysis performed was a single-channel analysis between the cases (SG2 and EV). To select candidate proteins, we considered a fold change ≥ |1.5| and *p*-value ≤ 0.05.

### 4.12. Clustering and Network Analysis

IPA (Ingenuity Systems, Qiagen, Redwood City, CA, USA) software was used to determine the functional networks and pathways associated with the differentially abundant proteins. The cutoff for considering significance in the proteins in the IPA CORE analysis was based on a fold change ≥ |1.5| and *p*-value ≤ 0.05; the human was considered as the model organism for annotations.

Network and canonical pathway enrichment analyses were performed filtering for all tissues, cell lines, and human species. Significant genes and proteins were input into the ToppFun web application (part of the ToppGene suite) to determine the functional enrichment of the genes and proteins lists [83].

### 4.13. Statistical Analysis

All statistical data was analyzed via GraphPad Prism 7.0 (GraphPad Software, La Jolla, CA, USA). One-way and two-way ANOVA tests were performed as per the requirement of the analysis * *p* ≤ 0.05, ** *p* ≤ 0.01, *** *p* ≤ 0.001, **** *p* ≤ 0.0001.

## 5. Conclusions

RBPMS knockout promoted cell proliferation, invasion, and increased the cisplatin resistance of ovarian cancer cells. RBPMS knockout promoted a senescence phenotype and altered ncRNAs and mRNA/proteins associated with remodeling of the tumor microenvironment, cell detoxification, RNA processing, cytoskeleton network, and cell integrity, among others. RBPMS and its regulated genes may be considered as diagnostic, prognostic, and/or response to therapy biomarkers in ovarian cancer. The potential role of RBPMS as a tumor suppressor gene in ovarian cancer should be further investigated.

## Figures and Tables

**Table 1:** Tissue array sample composition and classification.

Type of Ovarian Cancer	Num. of Patient	Num. of Samples	Average Age	Subgroups							
Normal Tissue	9	27	33	Grades							
				(-)	I	I-II	II	II-III	III		
				27	0	0	0	0	0		
				Stage							
				I	1a	1A	II	IIB	IC	IIIC	IV
				N/A	N/A	N/A	N/A	N/A	N/A	N/A	N/A
Clear Cell Carcinoma	5	18	49	Grades							
				(-)	I	I-II	II	II-III	III		
				18							
				Stage							
				I	1a	1A	II	IIB	IC	IIIC	IV
				15			3				
Serous Papillary Adenocarcinoma	47	138	48	Grades							
				(-)	I	I-II	II	II-III	III		
				2	22	0	33	4	77		
				Stage							
				I	1a	1A	II	IIB	IC	IIIC	IV
				39	3	6	33	3	9	36	9
Endometrioid Adenocarcinoma	4	12	51	Grades							
				(-)	I	I-II	II	II-III	III		
				0	0	0	11	1	0		
				Stage							
				I	1a	1A	II	IIB	IC	IIIC	IV
				12	0	0	0	0	0	0	0
Mucinous Adenocarcinoma	4	12	50	Grades							
				(-)	I	I-II	II	II-III	III		
				2	4	2	4	0	0		
				Stage							
				I	1a	1A	II	IIB	IC	IIIC	IV
				3	0	3	0	0	3	3	0

**Table 2:** Top five canonical pathways generated with the 655 significant deregulated RNAs in RBPMS SG2 vs. EV clones.

Ingenuity Canonical Pathways	log10 (*p*-Value)	Ratio	Number of Genes	Genes
Hepatic Fibrosis/Hepatic Stellate Cell Activation	7.54	0.111	21	*ACTA2*, *CCR5*, *COL11A2*, *COL12A1*, *COL1A1*, *COL6A1*, *COL7A1*, *CXCL8*, *FGF2*, *HGF*, *IFNGR1*, *IL4R*, *IL6R*, *KLF6*, *MET*, *MMP1*, *MYL7*, *PDGFD*, *RELB*, *SERPINE1*, *TIMP1*
Axonal Guidance Signaling	5.26	0.0635	31	*ADAM19*, *ADAMTS1*, *ADAMTS10*, *ADAMTS5*, *BMP5*, *DPYSL2*, *EFNB2*, *EPHA7*, *FZD2*, *GNG5*, *HHIP*, *ITGA2B*, *ITGB8*, *LRRC4C*, *MET*, *MMP1*, *MMP10*, *MMP16*, *MMP3*, *MYL7*, *NRP2*, *PAK3*, *PDGFD*, *PIK3CA*, *PLCD4*, *PLCE1*, *ROBO3*, *SDC2*, *SEMA4A*, *TUBB2B*, *UNC5B*
Hepatic Fibrosis Signaling Pathway	4.45	0.0631	26	*ACTA2*, *ACVR2B*, *CACNA1A*, *CACNB2*, *CACNG7*, *COL11A2*, *COL1A1*, *CXCL8*, *FGF2*, *FZD2*, *ITGA2B*, *ITGB8*, *LRP1*, *MAP2K3*, *MMP1*, *MYL7*, *MYLK*, *PDGFD*, *PIK3CA*, *PTEN*, *RELB*, *RHOH*, *RND3*, *SERPINE1*, *TCF7L1*, *TIMP1*
Inhibition of Matrix Metalloproteases	4.3	0.184	7	*LRP1*, *MMP1*, *MMP10*, *MMP16*, *MMP3*, *SDC2*, *TIMP1*
Tumor Microenvironment Pathway	4.15	0.0843	15	*COL1A1*, *CXCL8*, *FGF2*, *FGF20*, *FGF5*, *HGF*, *IL6R*, *MAP3K14*, *MMP1*, *MMP10*, *MMP16*, *MMP3*, *PDGFD*, *PIK3CA*, *RELB*

**Table 3:** Top 20 differentially expressed RNA transcripts in RBPMS SG2 vs. EV clones. Green: long-noncoding RNAs.

ID	Symbol	Gene Name	Fold Change	*p* Value
ENSG00000236333	TRHDE-AS1	TRHDE antisense RNA 1	102.584	1.55 × 10^−9^
ENSG00000139287	TPH2	Tryptophan hydroxylase 2	35.23	1.01 × 10^−7^
ENSG00000011465	DCN	Decorin	32.901	1.38 × 10^−8^
ENSG00000149968	MMP3	Matrix metallopeptidase 3	30.545	2.2 × 10^−7^
ENSG00000072657	TRHDE	Thyrotropin releasing hormone degrading enzyme	20.316	9.97 × 10^−8^
ENSG00000165092	ALDH1A1	Aldehyde dehydrogenase 1 family member A1	17.445	1.11 × 10^−9^
ENSG00000139329	LUM	Lumican	15.076	3 × 10^−10^
ENSG00000135046	ANXA1	Annexin A1	11.889	2.24 × 10^−8^
ENSG00000230426	LINC01036	Long intergenic non-protein coding RNA 1036	4.594	5.06 × 10^−10^
ENSG00000106366	SERPINE1	Serpin family E member 1	2.62	6.33 × 10^−7^
ENSG00000196562	SULF2	Sulfatase 2	−70.19	6.09 × 10^−20^
ENSG00000006047	YBX2	Y-box binding protein 2	−113.464	7.9 × 10^−23^
ENSG00000173727	LOC101927789	FAU, ubiquitin like and ribosomal protein S30 fusion pseudogene	−230.342	5.94 × 10^−20^
ENSG00000183770	FOXL2	Forkhead box L2	−262.198	4.99 × 10^−26^
ENSG00000170962	PDGFD	Platelet derived growth factor D	−597.324	3.21 × 10^−20^
ENSG00000135269	TES	Testin LIM domain protein	−701.647	5.83 × 10^−28^
ENSG00000204397	CARD16	Caspase recruitment domain family member 16	−1361.887	1.48 × 10^−32^
ENSG00000251095	LOC105377329	Uncharacterized LOC105377329	−1382.487	3.83 × 10^−28^
ENSG00000198795	ZNF521	Zinc finger protein 521	−1412.411	2.15 × 10^−27^
ENSG00000250337	PURPL	p53 upregulated regulator of p53 levels	−1852.805	3.08 × 10^−26^

**Table 4:** Biological role of the top 20 differentially abundant RNA transcripts in RBPMS SG2 and EV clones.

Symbol	Biological Role	Reference
ARRB1	A scaffold protein that participates in the agonist-mediated desensitization of G-protein-coupled receptors. Depending on the cancer type, it has been reported as an oncogene or tumor suppressor gene.	[39]
ARRB2	A scaffold protein that participates in the agonist-mediated desensitization of G-protein-coupled receptor. Increased in colorectal cancer, renal cell carcinoma, and glioblatoma.	[40]
CPT1A	Plays a critical role in increasing the fatty acid oxidation required for the cellular fuel demands in radioresistant and chemoresistant cancer cells.	[41]
STXBP2	Significantly expressed in hemophagocytic lymphohistiocytosis (HLH), a disease featuring severe hyperinflammation caused by the uncontrolled proliferation of activated lymphocytes and macrophages.	[42]
RRM2B	Plays a crucial role in DNA repair, DNA damage response, oxygen sensing, and apoptosis pathways. Highly amplified in multiple tumor types.	[43]
RAB27A	Belongs to a small GTPase superfamily (Rab family). Increased in many cancers. Governs a variety of oncogenic functions, including cell proliferation, motility, metastasis, and chemosensitivity.	[31]
ORC3	Highly conserved six-subunit protein complex essential for the initiation of DNA replication in eukaryotic cells.	[32]
CCDC90B	Presumably a mitochondrial protein characterized by the presence of a domain of unknown function DUF1640.	[33]
RRM2	A reductase that catalyzes the formation of deoxyribonucleotides from ribonucleotides. Its increased levels have been associated with cell proliferation, invasion, and migration; its downregulation induces apoptosis and G1 arrest.	[43]
KIF2C	Functions as a microtubule-dependent molecular motor. Acts like an oncogene in many cancer types, where it promotes cell proliferation, migration, invasion, and metastasis.	[34]
FSCN1	An actin-bundling protein that cross links F-actin microfilaments into tight, parallel bundles. Elevated FSCN1 levels have been correlated with aggressive clinical progression, poor prognosis, and poor survival outcomes in many cancer types.	[35]
PABPC4L	Possesses a critical role in RNA processing. It travels from the nucleus to the cytoplasm with mRNAs, increases eIF4F assembly at caps, forms closed-looped RNA, aids in the recruitment of ribosomal subunits to 5′ UTRs, and increases the reuse of translational machinery after polypeptide synthesis.	[44]
GSTM1	Plays a role in the detoxification of metabolites of environmental carcinogens and protecting hosts against cancer.	[45]
GSTM4	Similar GSTM1, it functions in the detoxification of electrophilic compounds, including carcinogens, therapeutic drugs, environmental toxins, and products of oxidative stress, via conjugation with glutathione.	[46]
FLNA	An actin-binding protein that crosslinks actin filaments and links actin filaments to membrane glycoprotein, which contributes to stabilizing the cytoskeleton network and supports cell integrity.	[47]
HSPA7	Transcribed in response to stress and plays a causal role in cancer initiation. HSPA7 is a poor prognostic biomarker in kidney and hepatocellular cancers.	[48]
HGF	A receptor of MET, which plays a role in cancer growth and metastasis. Activation of MET activates multiple cellular responses involved in cell survival, morphogenesis, adhesion, migration, breakdown of the extracellular matrix (ECM), and angiogenesis.	[49]
ERO1A	A hypoxia-induced endoplasmic reticulum oxidase that regulates the translation and folding of oxidized proteins. Its high expression is associated with poor prognosis in patients due to its promoting the cell proliferation and migration of cancer cells.	[36]
GARS1	Aminoacyl-tRNA synthetases that charge tRNAs with their cognate amino acids.	[50]

**Table 5:** Top 5 canonical pathways generated with the 111 differentially abundant proteins in RBPMS SG2 vs. EV clones.

Ingenuity Canonical Pathways	−log10 (*p*-Value)	Ratio	Number of Proteins	Proteins
LPS/IL-1 Mediated Inhibition of RXR Function	3.9	0.0332	7	ACSL1, ALDH2, CPT1A, GSTM1, GSTM4, HS2ST1, MGST1
Pyrimidine Deoxyribonucleotides De Novo Biosynthesis I	3.7	0.136	3	AK4, RRM2, RRM2B
Glutathione-mediated Detoxification	3.53	0.12	3	GSTM1, GSTM4, MGST1
Xenobiotic Metabolism PXR Signaling Pathway	3.47	0.0341	6	ALDH2, GSTM1, GSTM4, HS2ST1, MGST1, PRKAR1A

**Table 6:** Top 20 differentially abundant proteins in RBPMS SG2 vs. EV clones.

Accession	Symbol	Gene Name	Fold Change	*p* Value
P49407-2	ARRB1	Arrestin beta 1	3.586	1.67 × 10^−7^
P32121-5	ARRB2	Arrestin beta 2	3.586	1.67 × 10^−7^
P50416-2	CPT1A	Carnitine palmitoyltransferase 1A	2.864	1.93 × 10^−8^
M0R376	STXBP2	Syntaxin binding protein 2	2.43	1.55 × 10^−8^
Q7LG56-3	RRM2B	Ribonucleotide reductase regulatory TP53 inducible subunit M2B	2.24	1.09 × 10^−7^
P51159-2	RAB27A	RAB27A, member RAS oncogene family	2.201	1.59 × 10^−6^
Q9UBD5-3	ORC3	Origin recognition complex subunit 3	2.173	2.47 × 10^−7^
E9PKQ5	CCDC90B	Coiled-coil domain containing 90B	2.15	1.15 × 10^−7^
A0A286YFD6	RRM2	Ribonucleotide reductase regulatory subunit M2	2.132	3.06 × 10^−7^
Q5JR91	KIF2C	Kinesin family member 2C	2.097	1.83 × 10^−6^
Q16658	FSCN1	Fascin actin-bundling protein 1	−2.037	3.76 × 10^−6^
P0CB38	PABPC4L	Poly(A) binding protein cytoplasmic 4 like	−2.086	3.33 × 10^−6^
P09488-2	GSTM1	Glutathione S-transferase mu 1	−2.104	1.55 × 10^−6^
A0A0A0MR85	GSTM4	Glutathione S-transferase mu 4	−2.104	1.55 × 10^−6^
H0Y5F3	FLNA	Filamin A	−2.47	3.25 × 10^−8^
P48741	HSPA7	Heat shock protein family A (Hsp70) member 7	−2.491	1.41 × 10^−6^
P14210-6	HGF	Hepatocyte growth factor	−2.6	3.31 × 10^−8^
G3V2H0	ERO1A	Endoplasmic reticulum oxidoreductase 1 alpha	−2.603	1.85 × 10^−7^
P41250	GARS1	Glycyl-tRNA synthetase 1	−2.73	3.21 × 10^−7^
F5GYB7	RECQL	RecQ like helicase	−3.468	5.25 × 10^−8^

**Table 7:** Biological role of the top 20 differentially abundant proteins in RBPMS SG2 vs. EV clones.

Symbol	Biological Role	Reference
ARRB1	Scaffold protein that participates in the agonist-mediated desensitization of G-protein-coupled receptors. Depending on the cancer type, it has been reported as an oncogene or tumor suppressor gene.	[39]
ARRB2	Scaffold protein that participates in the agonist-mediated desensitization of G-protein-coupled receptor. Increased in colorectal cancer, renal cell carcinoma, and glioblastoma.	[40]
CPT1A	Plays a critical role in increasing the fatty acid oxidation required for the cellular fuel demands in radioresistant and chemoresistant cancer cells.	[41]
STXBP2	Significantly expressed in hemophagocytic lymphohistiocytosis (HLH), a disease of severe hyperinflammation caused by the uncontrolled proliferation of activated lymphocytes and macrophages.	[42]
RRM2B	Plays a crucial role in DNA repair, DNA damage response, oxygen sensing, and apoptosis pathways. It is highly amplified in multiple tumor types.	[43]
RAB27A	Belongs to a small GTPase superfamily (Rab family). Increased in many cancers. Governs a variety of oncogenic functions, including cell proliferation, cell motility, metastasis, and chemosensitivity.	[31]
ORC3	Highly conserved six-subunit protein complex essential for the initiation of DNA replication in eukaryotic cells.	[32]
CCDC90B	Presumably, a mitochondrial protein is characterized by the presence of a domain of unknown function DUF1640.	[33]
RRM2	This reductase catalyzes the formation of deoxyribonucleotides from ribonucleotides. Its increased levels have been associated with cell proliferation, invasion, and migration. Its downregulation induces apoptosis and G1 arrest.	[43]
KIF2C	Functions as a microtubule-dependent molecular motor. Acts like an oncogene in many cancer types, where it promotes cell proliferation, migration, invasion, and metastasis.	[34]
FSCN1	An actin-bundling protein that cross-links F-actin microfilaments into tight, parallel bundles. Elevated FSCN1 levels have been correlated with aggressive clinical progression, poor prognosis, and poor survival outcomes in many cancer types.	[35]
PABPC4L	Has a critical role in RNA processing. It travels from the nucleus to the cytoplasm with mRNAs, increases eIF4F assembly at caps, forms closed-looped RNA, aids in the recruitment of ribosomal subunits to 5′ UTRs, and increases the reuse of translational machinery after polypeptide synthesis.	[44]
GSTM1	Plays a role in the detoxification of metabolites of environmental carcinogens and protects hosts against cancer.	[54]
GSTM4	Similar to GSTM1, functions in the detoxification of electrophilic compounds, including carcinogens, therapeutic drugs, environmental toxins, and products of oxidative stress, via conjugation with glutathione.	[46]
FLNA	An actin-binding protein that crosslinks actin filaments and links actin filaments to membrane glycoproteins, contributing to stabilizing the cytoskeleton network and supporting cell integrity.	[47]
HSPA7	Transcribed in response to stress and plays a causal role in cancer initiation. HSPA7 is a poor prognostic biomarker in kidney and hepatocellular cancers.	[48]
HGF	A receptor of MET, which play a role in cancer growth and metastasis. The activation of MET activates multiple cellular responses involved in cell survival, morphogenesis, adhesion, migration, breakdown of the extracellular matrix (ECM), and angiogenesis.	[49]
ERO1A	Is a hypoxia-induced endoplasmic reticulum oxidase that regulates the translation and folding of oxidized proteins. The high expression of ERO1A is associated with poor prognosis in patients by its promoting cell proliferation and the migration of cancer cells.	[36]
GARS1	Aminoacyl-tRNA synthetases that charge tRNAs with their cognate amino acids.	[50]

**Table 8:** Common differentially abundant genes at the RNA and protein levels in RBPMS SG2 vs. EV clones.

Gene Symbol	Uniprot	Gene Name	Fold Change RNAseq	*p*-Value RNAseq	Fold Change Proteomics	*p*-Values Proteomics
ANXA1	Q5T3N1	Annexin A1	11.889	2.24 × 10^−08^	3.047	4.59 × 10^−5^
FDFT1	E9PJG4	Farnesyl-diphosphate farnesyltransferase 1	6.7	2.03 × 10^−06^	2.587	3.30 × 10^−6^
TIMP1	Q5H9A7	TIMP metallopeptidase inhibitor 1	2.637	4.83 × 10^−3^	2.456	3.55 × 10^−3^
SSX2IP	S4R403	SSX family member 2 interacting protein	2.406	5.83 × 10^−4^	2.009	9.71 × 10^−6^
SPART	Q8N0X7	Spartin	1.738	7.7 × 10^−4^	2.046	9.79 × 10^−5^
CBS/CBSL	H7C2W0	Cystathionine beta-synthase	−1.517	8.02 × 10^−3^	−2.314	2.92 × 10^−4^
GSTM1	P09488-2	Glutathione S-transferase mu 1	−1.643	6.72 × 10^−4^	−2.104	1.55 × 10^−6^
HGF	P14210-6	Hepatocyte growth factor	−1.767	2.93 × 10^−3^	−2.6	3.31 × 10^−8^
UACA	F5H2B9	Uveal autoantigen with coiled-coil domains and ankyrin repeats	−2.451	2.08 × 10^−4^	−2.212	2.44 × 10^−3^
ACTN3	Q08043	Actinin alpha 3	−2.455	4.93 × 10^−4^	−2.091	1.92 × 10^−5^

## Data Availability

Not applicable.

## Supplementary Materials

The following are available online at https://www.mdpi.com/article/10.3390/ijms23010535/s1.
