# Supplementary material for: Reduced RBPMS Levels Promote Cell Proliferation and Decrease Cisplatin Sensitivity in Ovarian Cancer Cells
Source: Int J Mol Sci. 2022 Jan 4;23(1):535. doi: 10.3390/ijms23010535 (PMC8745614; doi:10.3390/ijms23010535)
Supplement: Supplementary file 1 [file ijms-23-00535-s001.zip › ijms-1512411-supplementary/data suplementaria sometida/Supplementary figures and tables.pptx]

## Slide 1
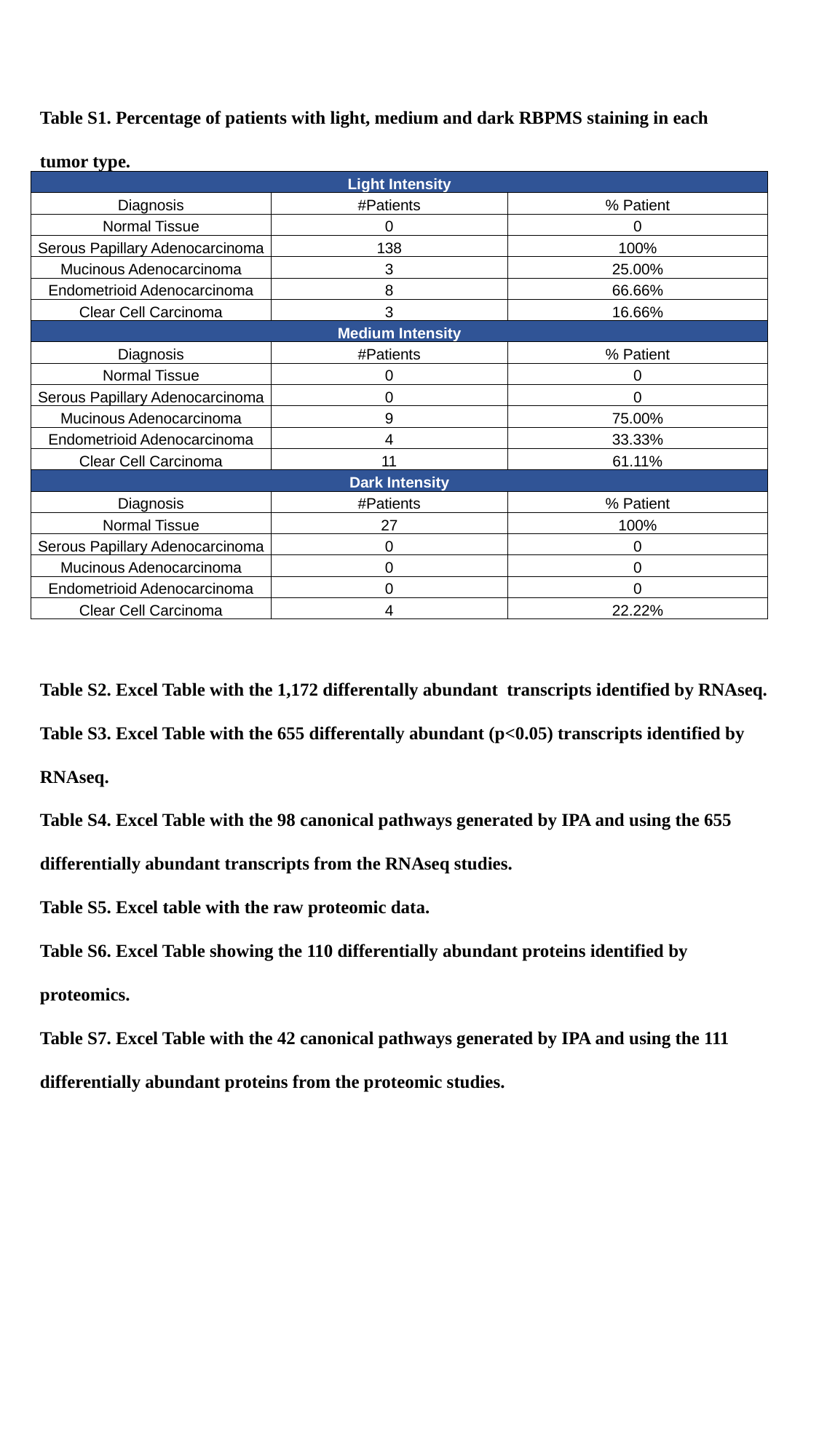

Table S1. Percentage of patients with light, medium and dark RBPMS staining in each tumor type.
| Light Intensity | | |
| --- | --- | --- |
| Diagnosis | #Patients | % Patient |
| Normal Tissue | 0 | 0 |
| Serous Papillary Adenocarcinoma | 138 | 100% |
| Mucinous Adenocarcinoma | 3 | 25.00% |
| Endometrioid Adenocarcinoma | 8 | 66.66% |
| Clear Cell Carcinoma | 3 | 16.66% |
| Medium Intensity | | |
| Diagnosis | #Patients | % Patient |
| Normal Tissue | 0 | 0 |
| Serous Papillary Adenocarcinoma | 0 | 0 |
| Mucinous Adenocarcinoma | 9 | 75.00% |
| Endometrioid Adenocarcinoma | 4 | 33.33% |
| Clear Cell Carcinoma | 11 | 61.11% |
| Dark Intensity | | |
| Diagnosis | #Patients | % Patient |
| Normal Tissue | 27 | 100% |
| Serous Papillary Adenocarcinoma | 0 | 0 |
| Mucinous Adenocarcinoma | 0 | 0 |
| Endometrioid Adenocarcinoma | 0 | 0 |
| Clear Cell Carcinoma | 4 | 22.22% |
Table S2. Excel Table with the 1,172 differentally abundant transcripts identified by RNAseq.
Table S3. Excel Table with the 655 differentally abundant (p<0.05) transcripts identified by RNAseq.
Table S4. Excel Table with the 98 canonical pathways generated by IPA and using the 655 differentially abundant transcripts from the RNAseq studies.
Table S5. Excel table with the raw proteomic data.
Table S6. Excel Table showing the 110 differentially abundant proteins identified by proteomics.
Table S7. Excel Table with the 42 canonical pathways generated by IPA and using the 111 differentially abundant proteins from the proteomic studies.

## Slide 2
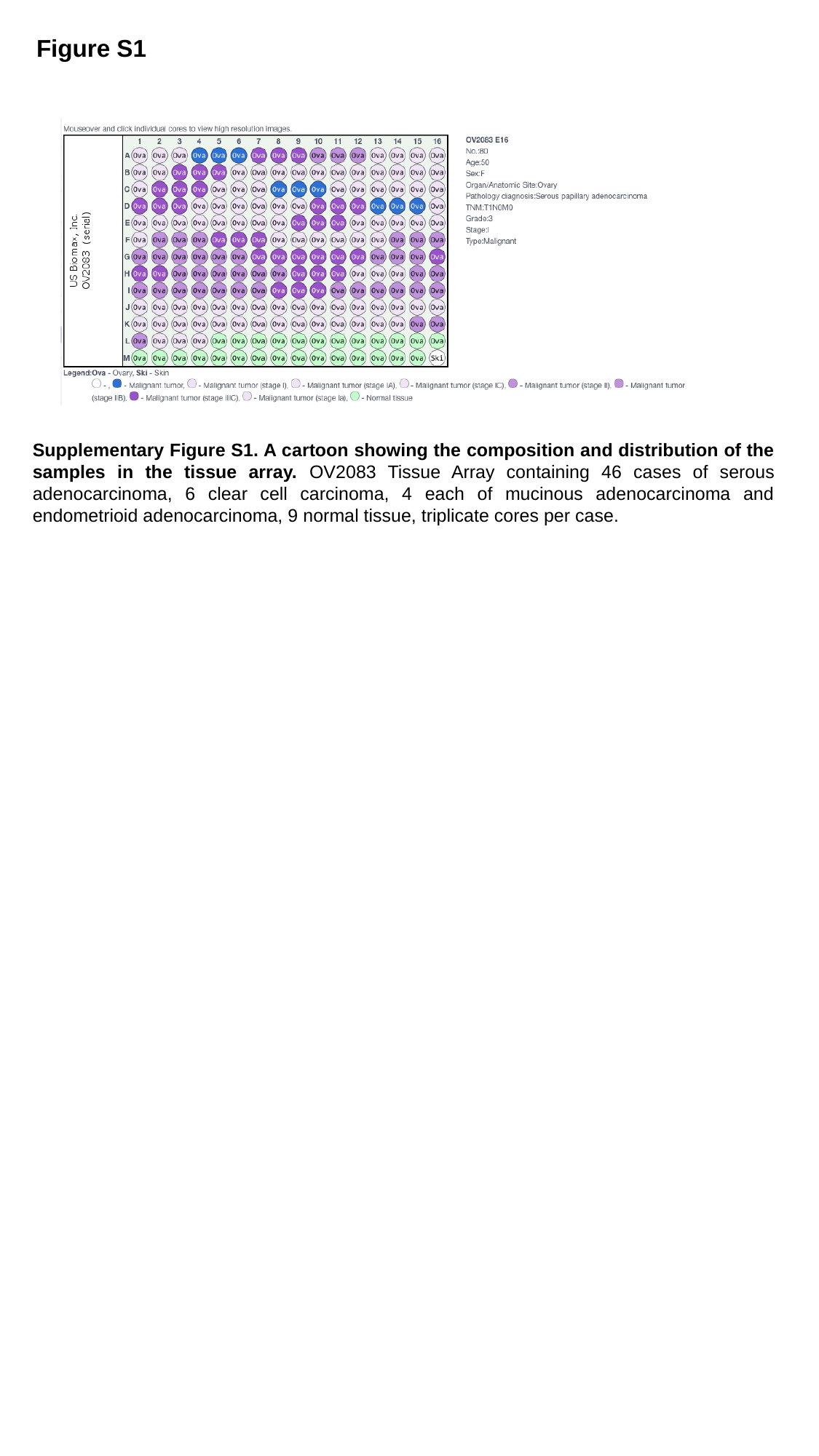

Figure S1
Supplementary Figure S1. A cartoon showing the composition and distribution of the samples in the tissue array. OV2083 Tissue Array containing 46 cases of serous adenocarcinoma, 6 clear cell carcinoma, 4 each of mucinous adenocarcinoma and endometrioid adenocarcinoma, 9 normal tissue, triplicate cores per case.

## Slide 3
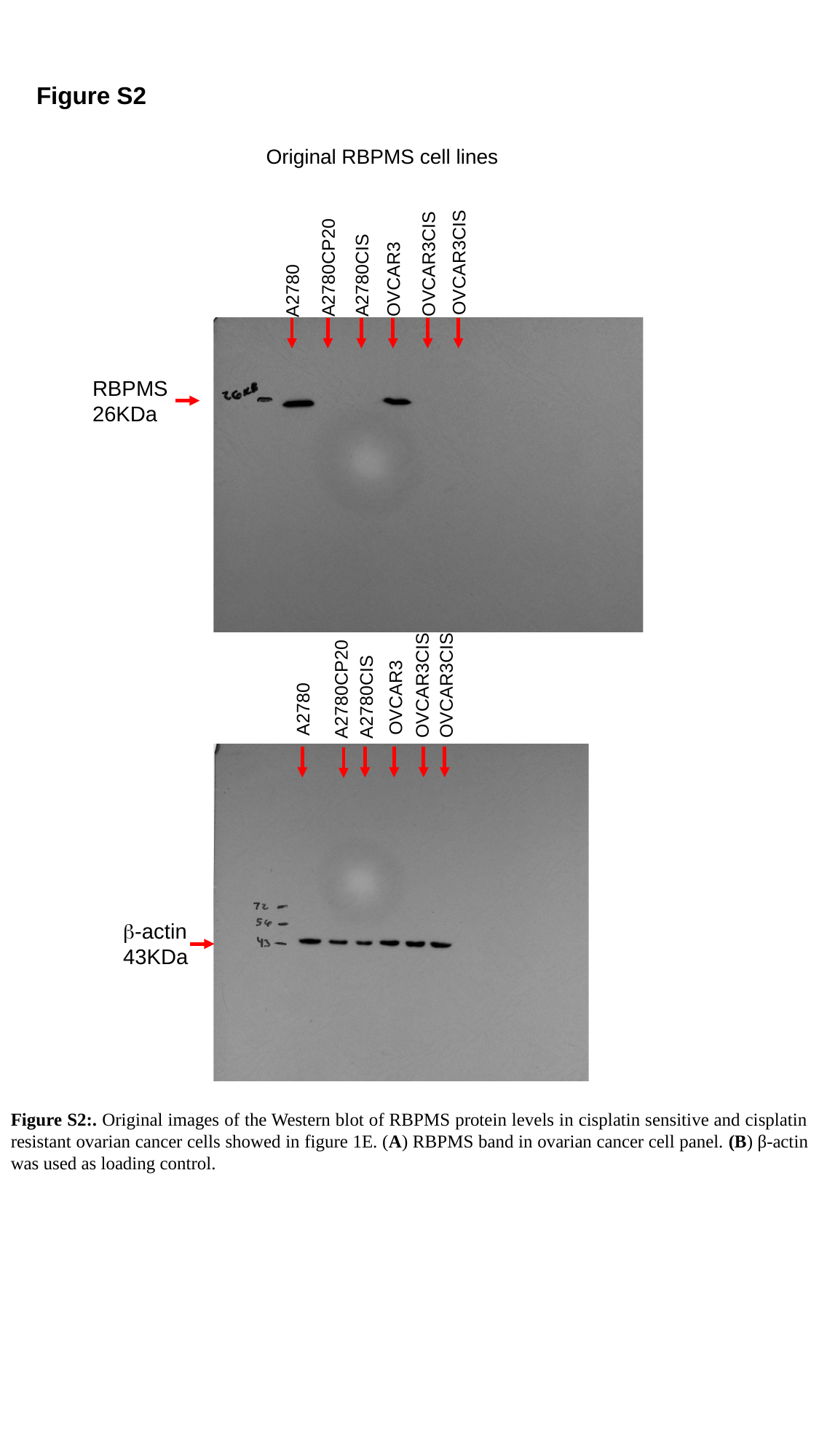

Figure S2
Original RBPMS cell lines
OVCAR3CIS
OVCAR3CIS
A2780CP20
A2780CIS
OVCAR3
A2780
RBPMS
26KDa
OVCAR3CIS
OVCAR3CIS
A2780CP20
A2780CIS
OVCAR3
A2780
β-actin
43KDa
Figure S2:. Original images of the Western blot of RBPMS protein levels in cisplatin sensitive and cisplatin resistant ovarian cancer cells showed in figure 1E. (A) RBPMS band in ovarian cancer cell panel. (B) β-actin was used as loading control.

## Slide 4
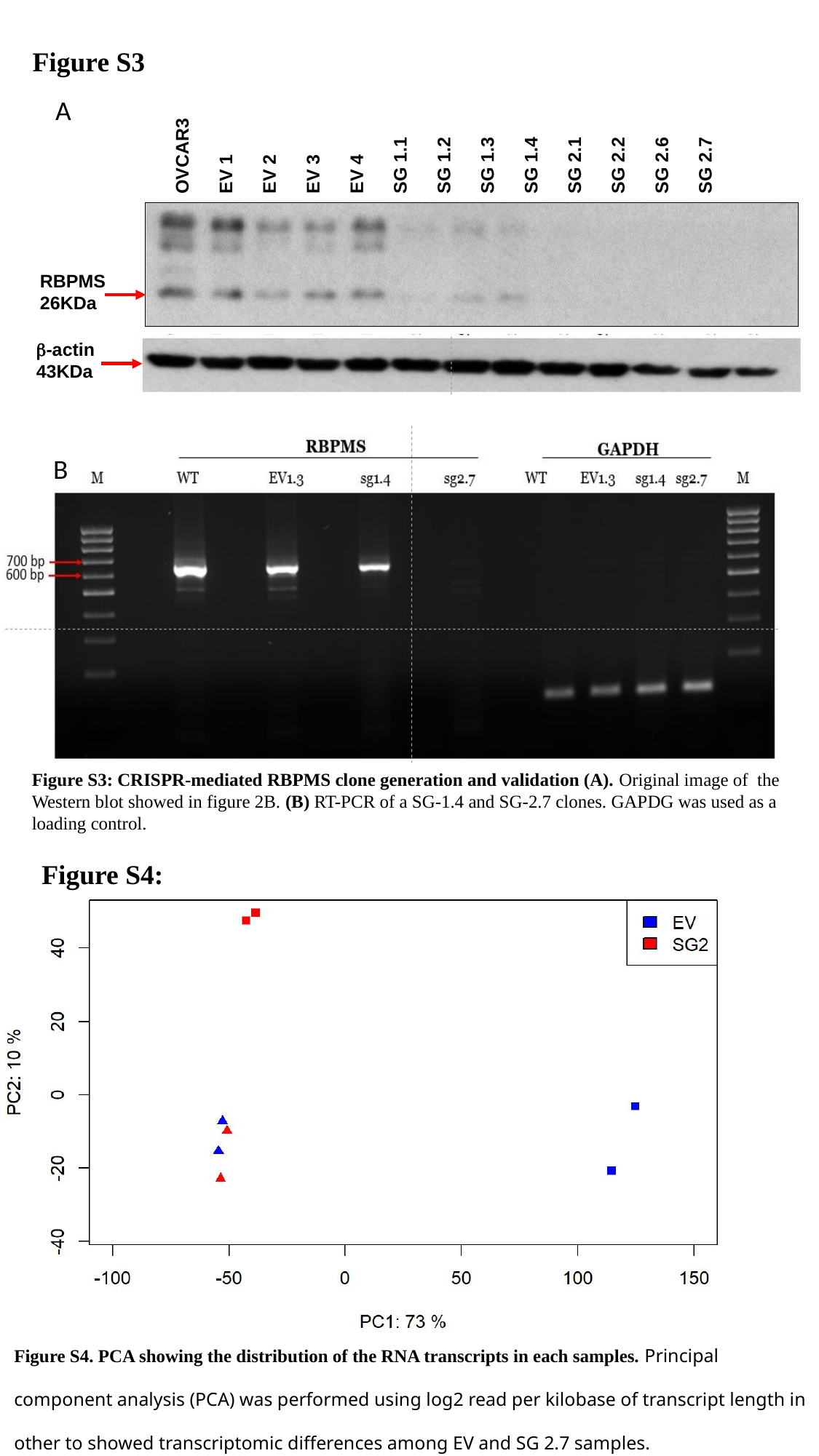

OVCAR3
EV 1
EV 2
EV 3
EV 4
SG 1.1
SG 1.2
SG 1.3
SG 1.4
SG 2.1
SG 2.2
SG 2.6
SG 2.7
Figure S3
A
RBPMS
26KDa
β-actin
43KDa
B
Figure S3: CRISPR-mediated RBPMS clone generation and validation (A). Original image of the Western blot showed in figure 2B. (B) RT-PCR of a SG-1.4 and SG-2.7 clones. GAPDG was used as a loading control.
Figure S4:
Figure S4. PCA showing the distribution of the RNA transcripts in each samples. Principal component analysis (PCA) was performed using log2 read per kilobase of transcript length in other to showed transcriptomic differences among EV and SG 2.7 samples.

## Slide 5
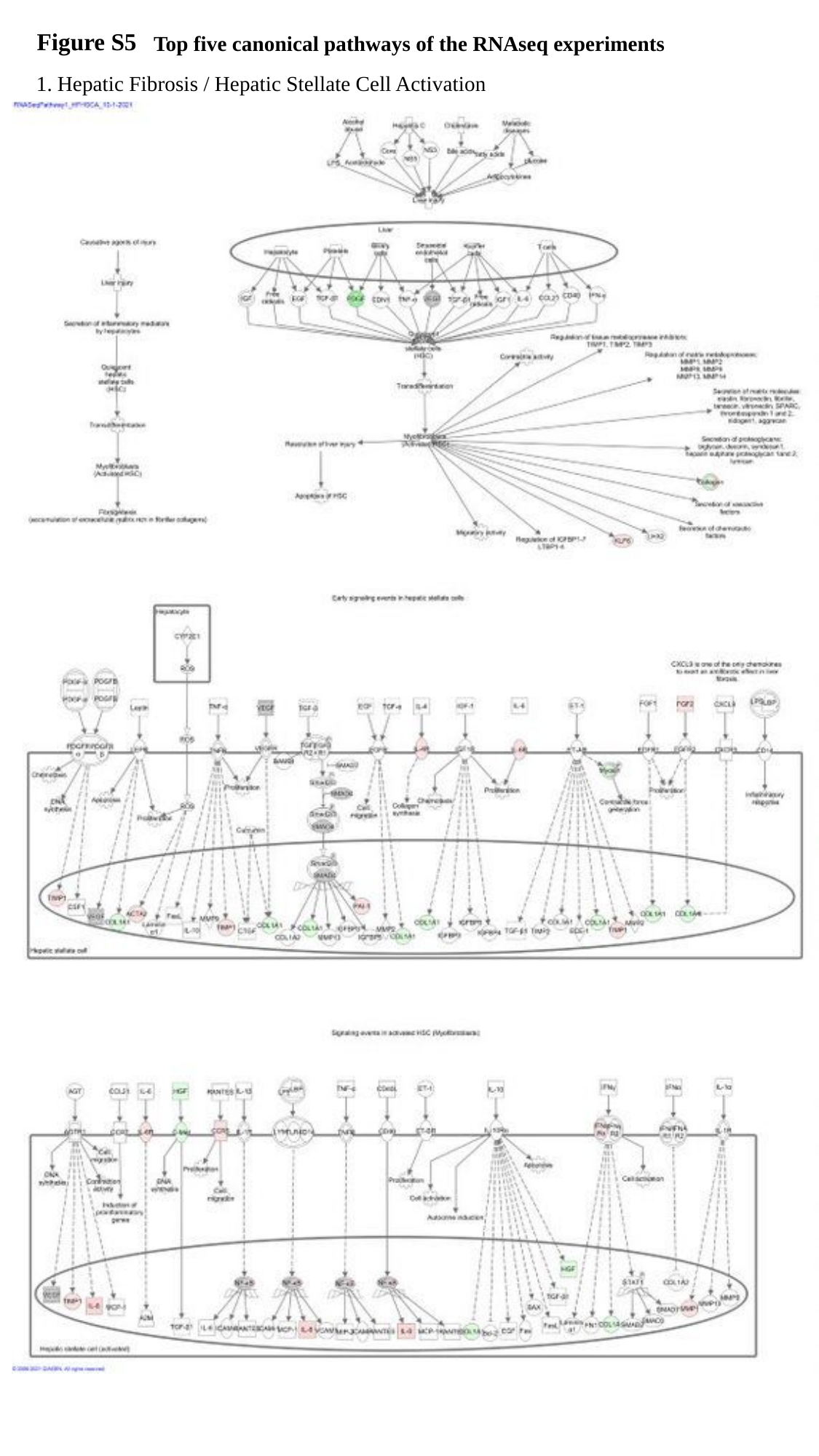

Figure S5
Top five canonical pathways of the RNAseq experiments
1. Hepatic Fibrosis / Hepatic Stellate Cell Activation

## Slide 6
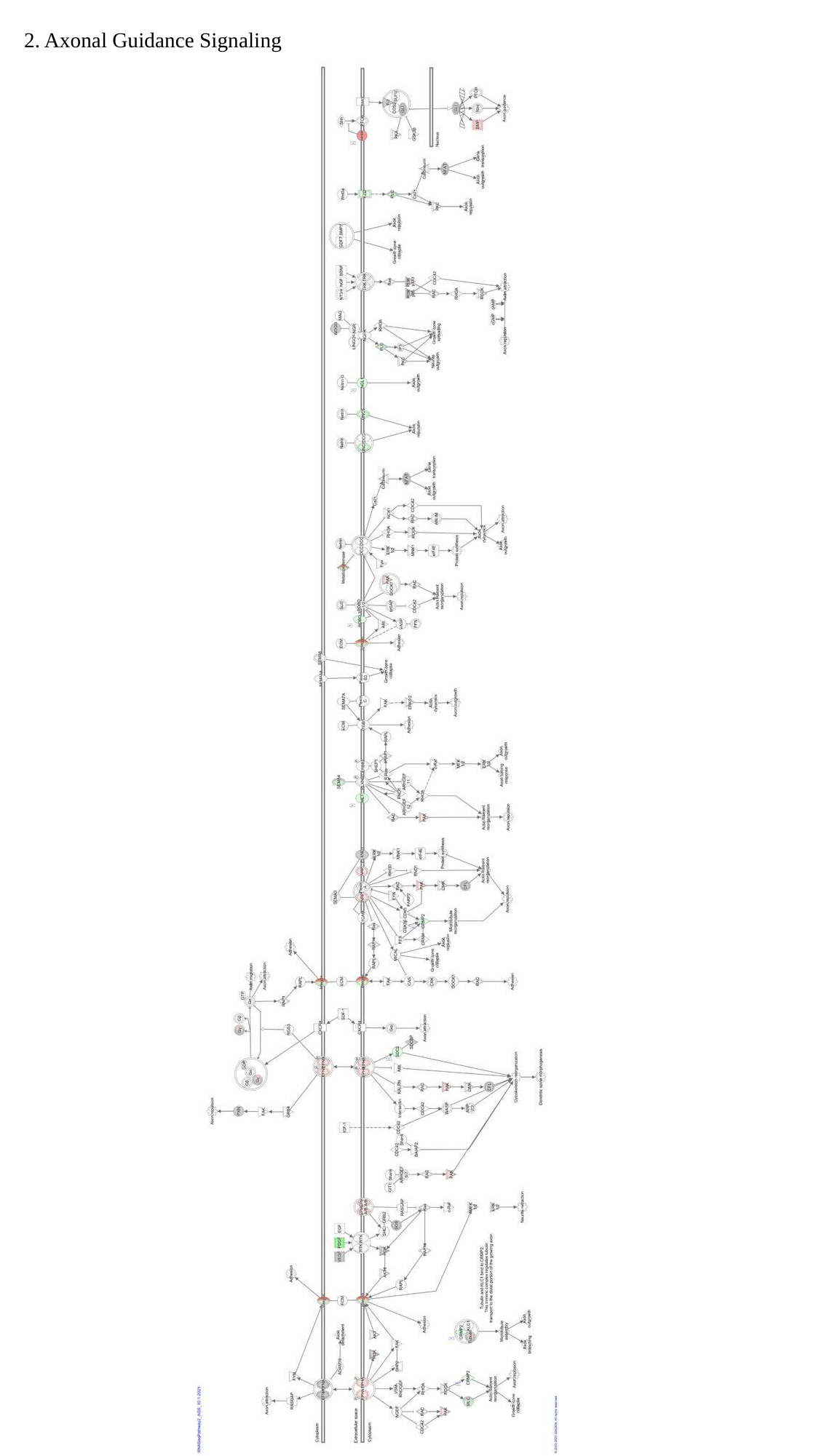

2. Axonal Guidance Signaling

## Slide 7
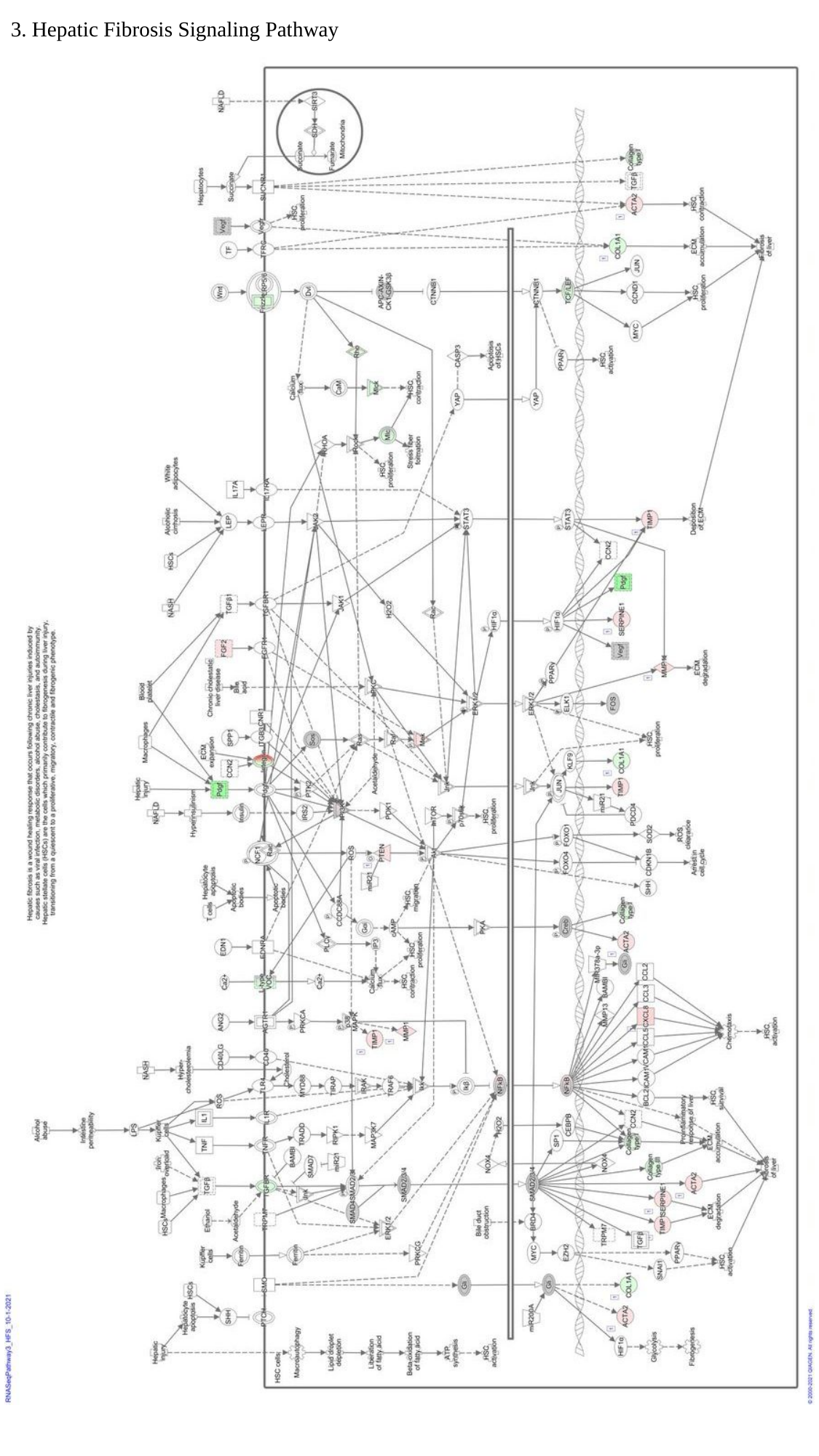

3. Hepatic Fibrosis Signaling Pathway

## Slide 8
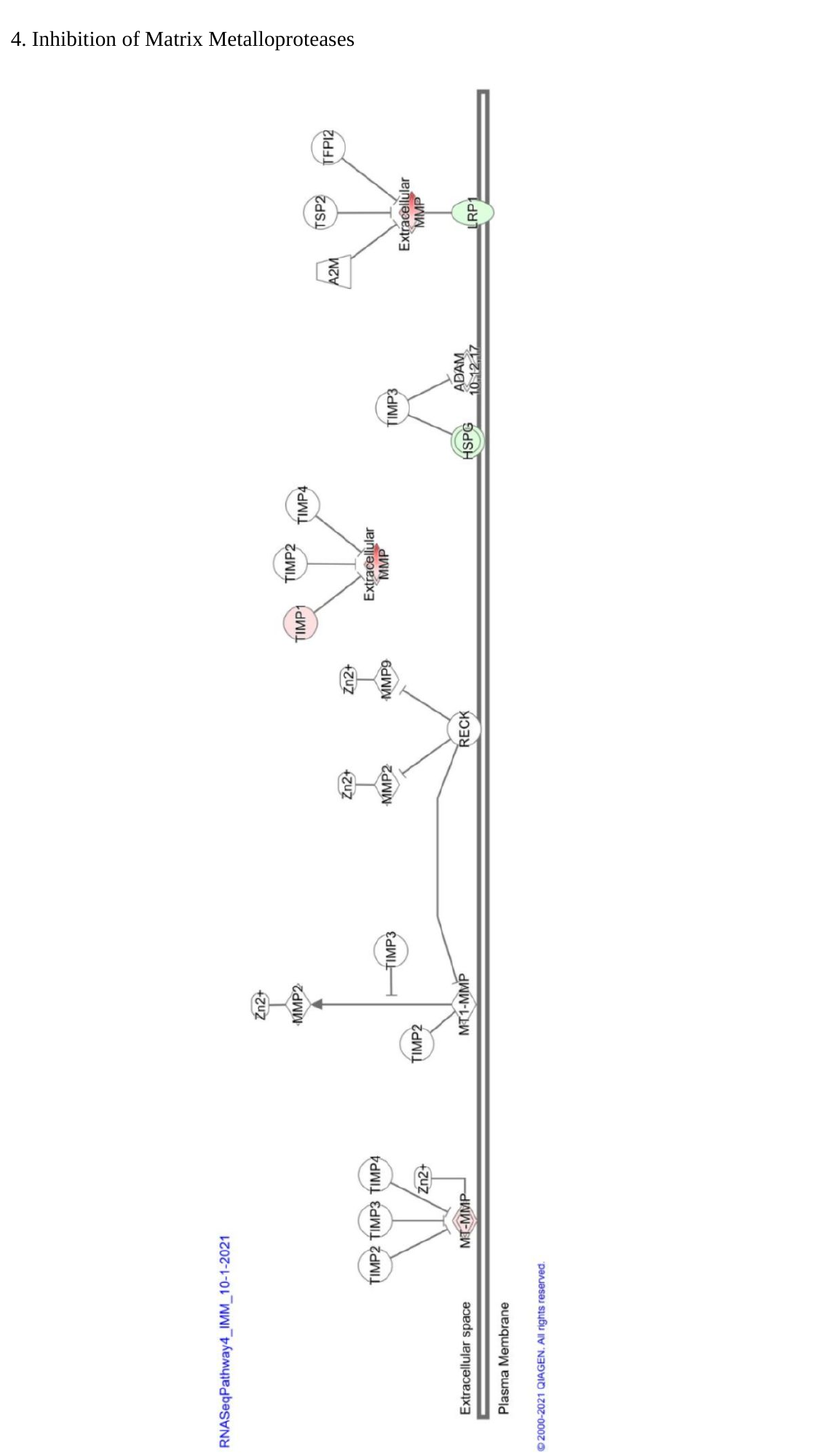

4. Inhibition of Matrix Metalloproteases

## Slide 9
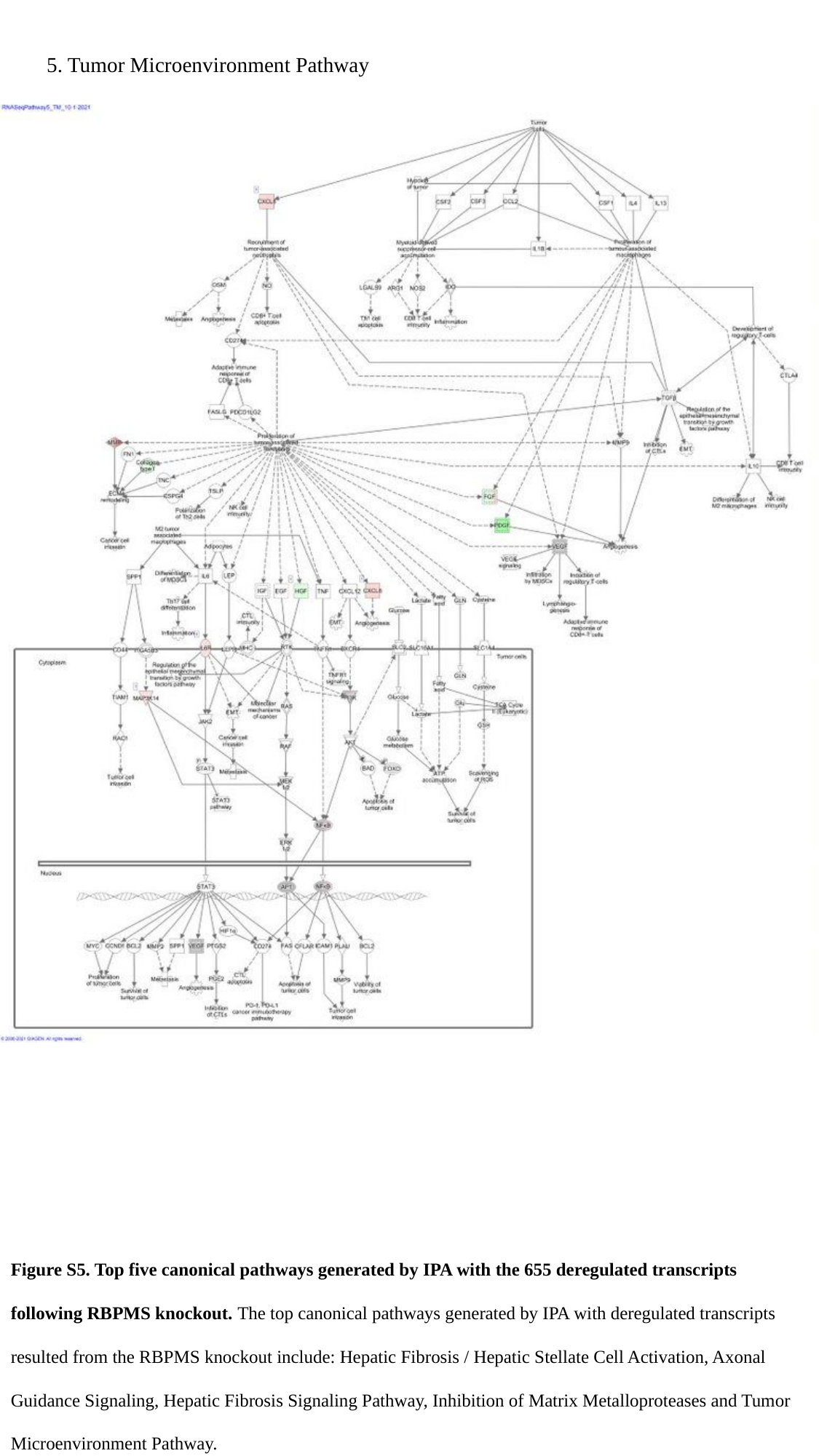

5. Tumor Microenvironment Pathway
Figure S5. Top five canonical pathways generated by IPA with the 655 deregulated transcripts following RBPMS knockout. The top canonical pathways generated by IPA with deregulated transcripts resulted from the RBPMS knockout include: Hepatic Fibrosis / Hepatic Stellate Cell Activation, Axonal Guidance Signaling, Hepatic Fibrosis Signaling Pathway, Inhibition of Matrix Metalloproteases and Tumor Microenvironment Pathway.

## Slide 10
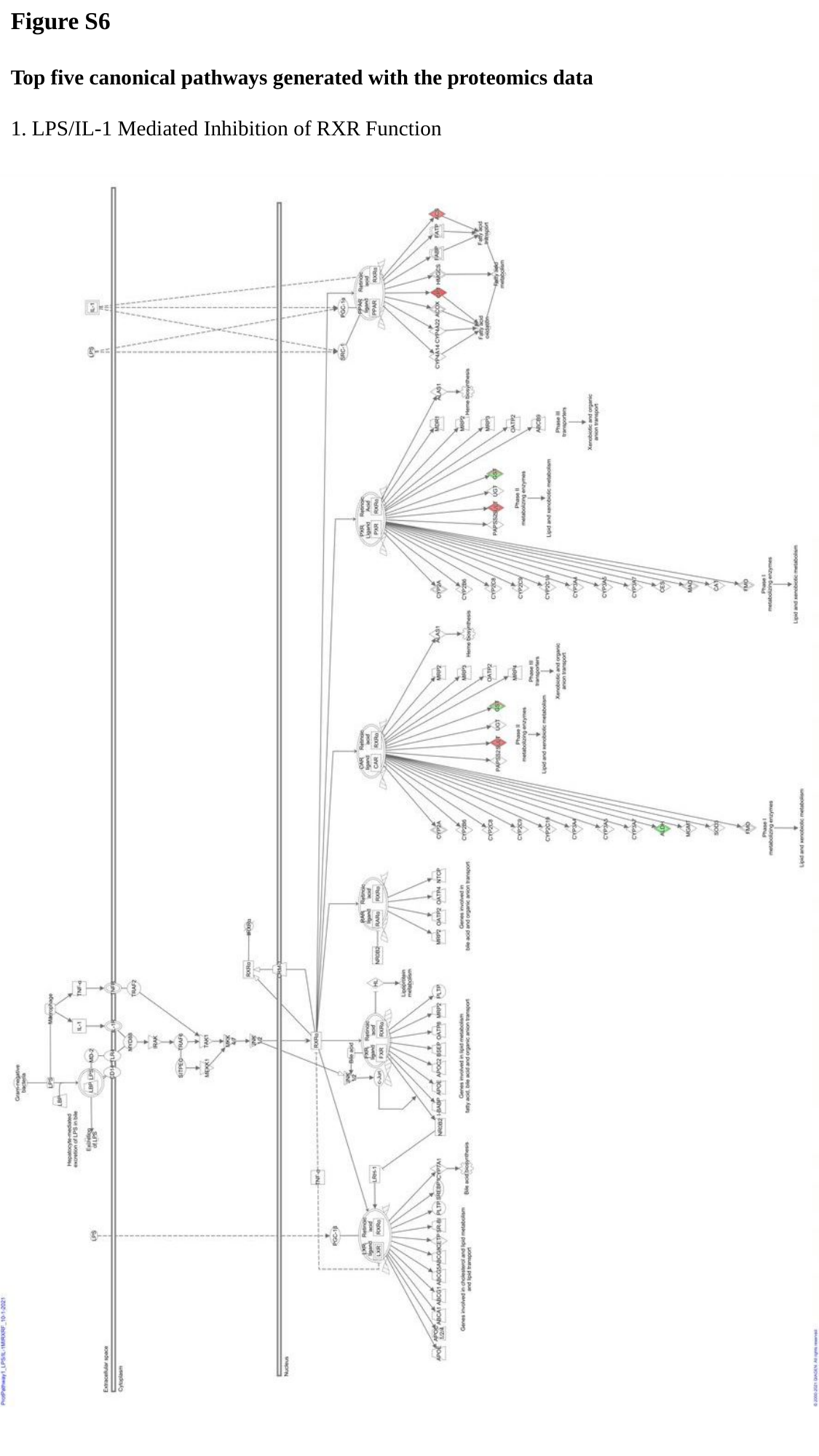

Figure S6
Top five canonical pathways generated with the proteomics data
1. LPS/IL-1 Mediated Inhibition of RXR Function

## Slide 11
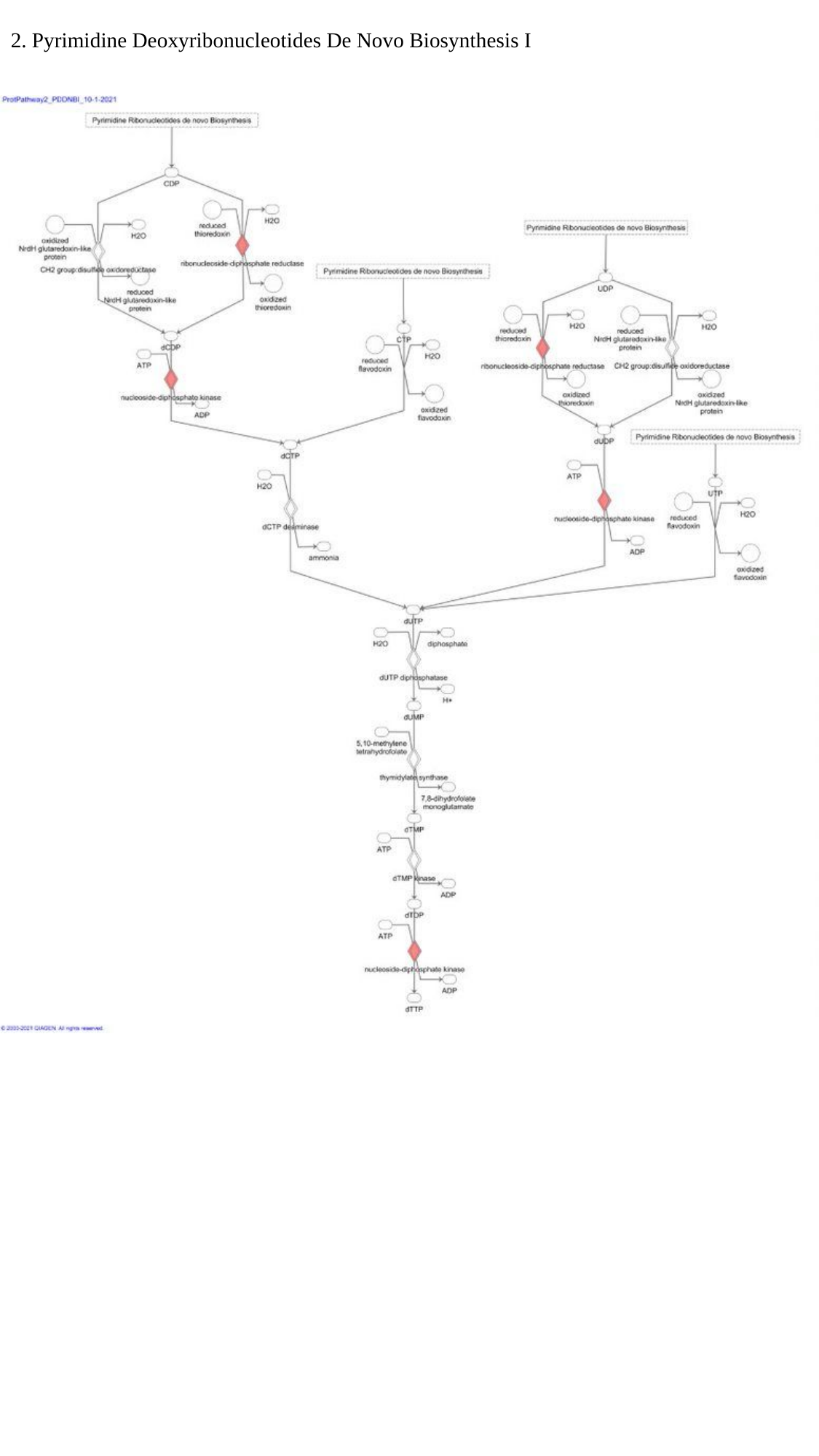

2. Pyrimidine Deoxyribonucleotides De Novo Biosynthesis I

## Slide 12
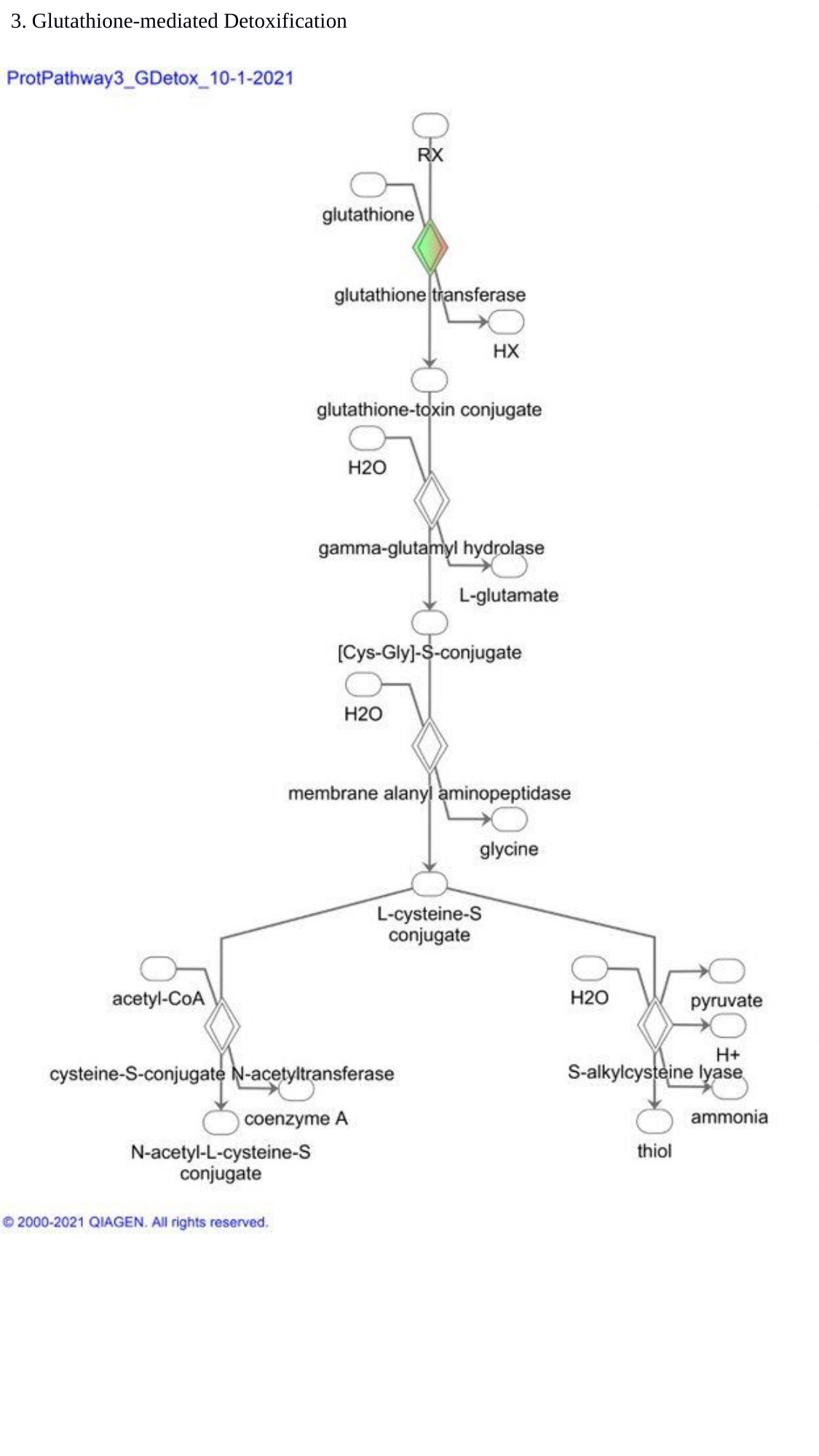

3. Glutathione-mediated Detoxification

## Slide 13
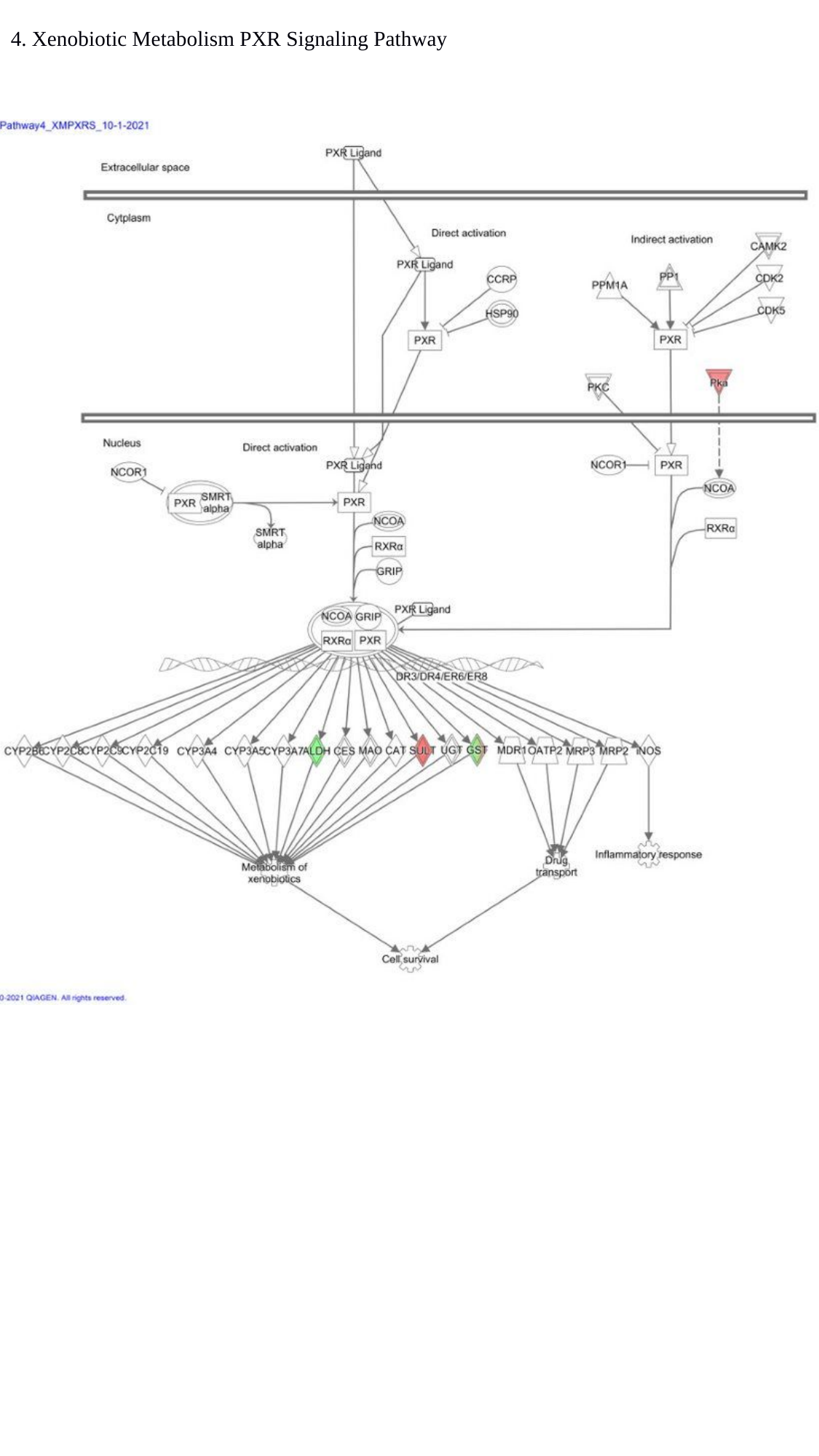

4. Xenobiotic Metabolism PXR Signaling Pathway

## Slide 14
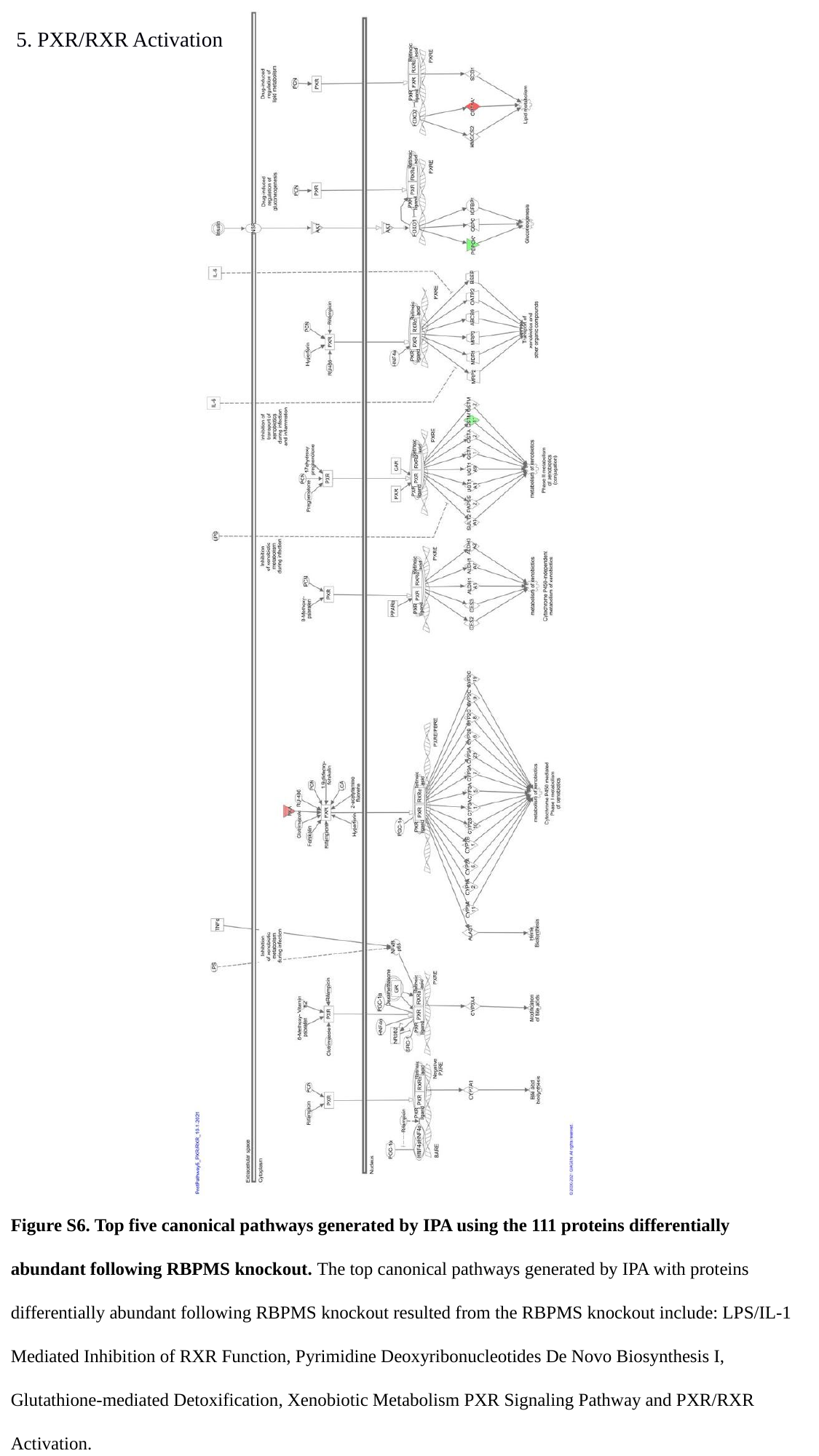

5. PXR/RXR Activation
Figure S6. Top five canonical pathways generated by IPA using the 111 proteins differentially abundant following RBPMS knockout. The top canonical pathways generated by IPA with proteins differentially abundant following RBPMS knockout resulted from the RBPMS knockout include: LPS/IL-1 Mediated Inhibition of RXR Function, Pyrimidine Deoxyribonucleotides De Novo Biosynthesis I, Glutathione-mediated Detoxification, Xenobiotic Metabolism PXR Signaling Pathway and PXR/RXR Activation.

## Slide 15
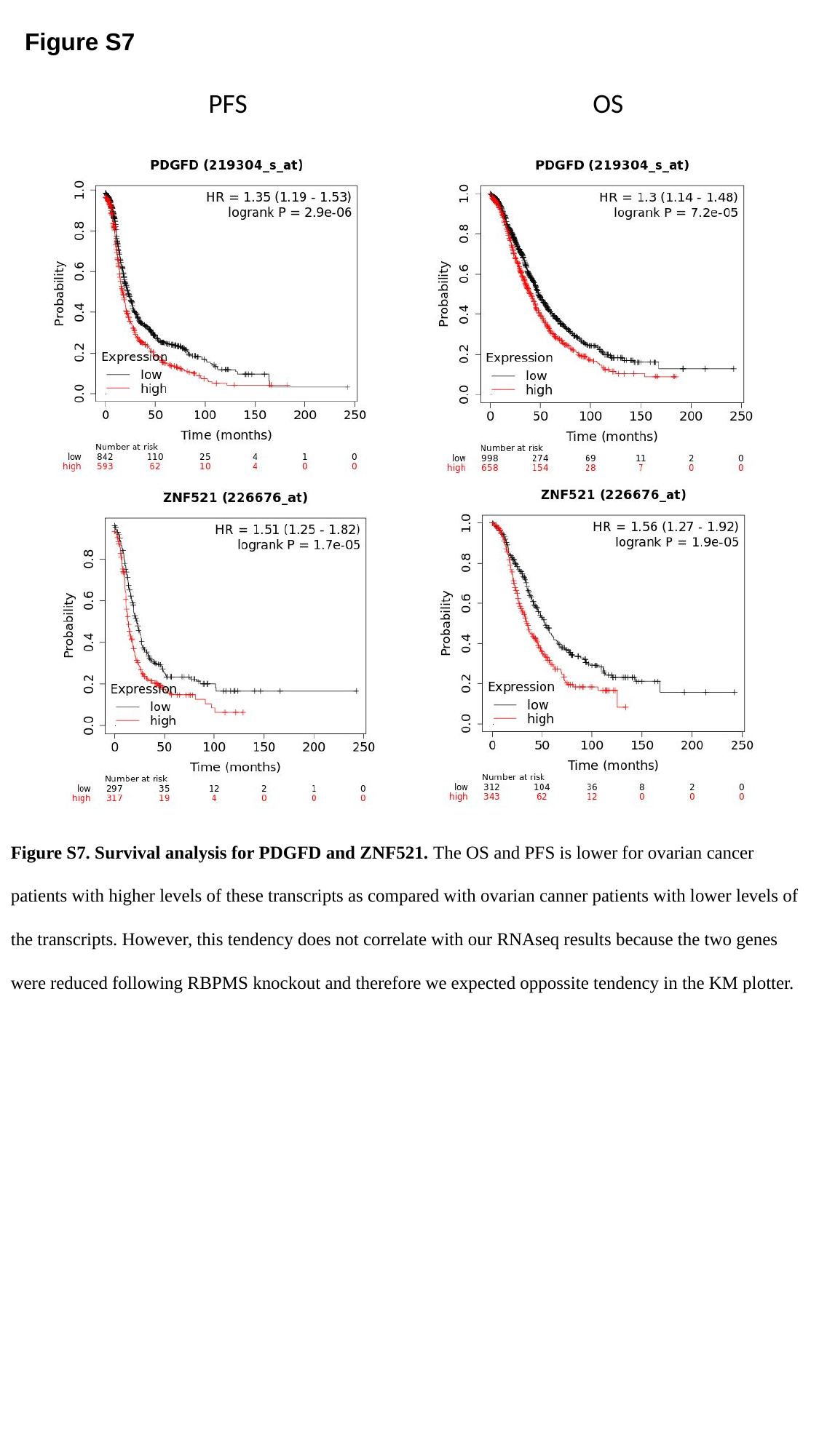

Figure S7
 PFS OS
Figure S7. Survival analysis for PDGFD and ZNF521. The OS and PFS is lower for ovarian cancer patients with higher levels of these transcripts as compared with ovarian canner patients with lower levels of the transcripts. However, this tendency does not correlate with our RNAseq results because the two genes were reduced following RBPMS knockout and therefore we expected oppossite tendency in the KM plotter.

## Slide 16
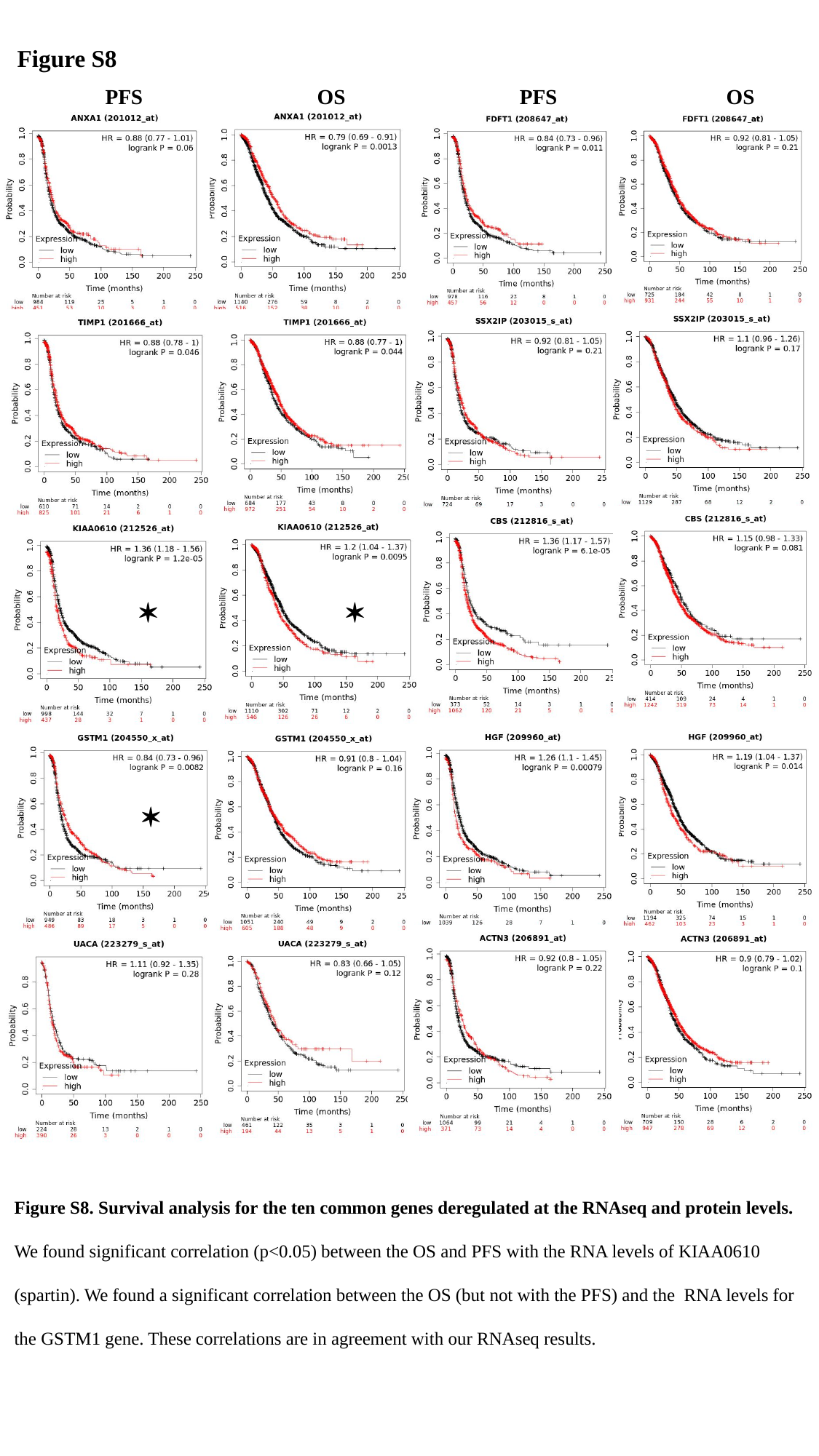

Figure S8
PFS OS PFS OS



Figure S8. Survival analysis for the ten common genes deregulated at the RNAseq and protein levels. We found significant correlation (p<0.05) between the OS and PFS with the RNA levels of KIAA0610 (spartin). We found a significant correlation between the OS (but not with the PFS) and the RNA levels for the GSTM1 gene. These correlations are in agreement with our RNAseq results.
